# Limitation of convergence-confinement method on three-dimensional tunnelling effect

**DOI:** 10.1038/s41598-023-29062-5

**Published:** 2023-02-03

**Authors:** Liuming Chang, Leandro R. Alejano, Lan Cui, Qian Sheng, Mingxing Xie

**Affiliations:** 1 College of Civil Engineering and Architecture, Anhui University of Science and Technology, Huainan, 232001 China; 2 CINTECX, Department of Natural Resources and Environmental Engineering, University of Vigo, Vigo, Spain; 3 State Key Laboratory of Geomechanics and Geotechnical Engineering, Institute of Rock and Soil Mechanics, Chinese Academy of Sciences, Wuhan, 430071 China; 4 University of Chinese Academy of Sciences, Beijing, 100049 China; 5 College of Civil Engineering, Taiyuan University of Technology, Taiyuan, 030024 China

**Keywords:** Civil engineering, Petrology

## Abstract

The convergence-confinement method (CCM) is a simplified widely utilised tool for assessing the interplay between the rock mass behaviour and the support effect, so it is quite helpful for tunnel support design purposes. However, the direct application of this technique has shown some limitations, many of which are directly related to the three-dimensionality issue. Indeed, the CCM tries to solve the three-dimensional (3D) problem of tunnel advance deformation and support response, by means of a series of two-dimensional (2D) plane strain analyses. So, regardless purely elastic cases, certain deviation is observed when comparing CCM and 3D numerical modelling results. The reasons behind this deviation have been studied from different points of views, but they seem to be still not well understood. With the aim of advancing towards a better knowledge of this issue, this paper discusses the limitation of CCM to correctly reflect the 3D tunnelling effect by comparing CCM and 3D numerical deformation, support pressure and liner load results in a typical tunnel case for various geological conditions. The reasons for CCM results in different rock deformation and support pressure in comparing to the 3D numerical modelling are explained. Some guidelines are eventually given recommending when the use of CCM can be acceptable according to the rock mass strength and tunnel depth, and when a more rigorous 3D approach is convenient.

## Introduction

The support design of the underground openings has always been considered as a highly complicated issue from the view of practical engineering. The numerical and analytical models are proved to be powerful in obtaining a better understanding of the interaction between the rock mass and the support. From the standpoint of engineering, the models need to be simplified and convenient. The convergence-confinement method (CCM) is such an appropriate model due to its simplification of evaluating the rock mass behaviour in relation to the support design^1–3^. CCM is composed of three curves: the ground reaction curve (GRC), the longitudinal deformation profile (LDP) and the support characteristic curve (SCC). It solves out the interaction process that takes place close to the tunnel face by intersecting these three curves. Although the method is two-dimensional (2D), its result is applied to the three-dimensional (3D) problem because CCM not only illustrates the rock mass and support in the transverse section but implies the advance of the tunnel face and installation of the support in the longitudinal direction.

Despite of the advantages of the CCM, there are some limitations that restrict its application. For example, the method has difficulty in properly simulating some internal support types like rock bolts, forepole umbrellas or composite supports for instance, those composed by a concrete ring and rock bolts. CCM commonly applies to a full-face method, but cannot be applied to sequenced excavation methods. Moreover, the complicated stress–strain relations of the rock mass such as the rheological and strain-softening behaviours are not comprehensively reflected in the method. To overcome the limitations, some researchers modified certain components of the CCM such as LDP, GRC, or SCC based on numerical or analytical results. Carranza-Torres and Engen^4^ constructed the SCC of a combined support system consisting of closed circular steel sets with equally spaced prismatic wood blocks. Gonzalez-Cao et al.^5^ included the blast damage to the rock mass in GRC by assigning different disturbance factors. Paraskevopoulou and Diederichs^6^, Song et al.^7^ proposed new LDPs that considered the rheological behaviour. Ranjbarnia et al.^8^ presented a nonlinear SCC by introducing a qualitative distribution of the load on the arch umbrella elements. Vlachopoulos and Diederichs^9^ extrapolated the CCM to apply staged excavation sequences by modifying the LDP. Gonzalez-Nicieza et al.^10^ considered the tunnel depth and the shape of the cross-section of the tunnel by modifying several variables in the LDP on the basis of the 3D simulations. Alejano et al.^11–13^ and Cui et al.^14^ took account of the strain-softening behaviour of the rock mass by proposing procedures for obtaining the corresponding GRC.

The above analyses focused on improving the CCM based on the details of support types and rock mass behaviours. Essentially, one of the most important limitations of the CCM is related to the difficulties in correctly representing the actual three-dimensional behaviour of the tunnel problem^5,11,15,16^: although CCM tries to obtain the 3D problem by means of a series of 2D plane strain analysis, significant deviation is often observed by comparing the results of CCM with the 3D numerical modelling. Due to this, several researchers placed more emphasis on the discrepancies between CCM and 3D numerical modelling. Zhao et al.^17^ applied the CCM and 3D numerical modelling to a hydroelectric project in India, but paid little attention to the reason why such deviations occur. Cantieni and Anagnostou^18,19^, Alejano et al.^11^and Fuente et al.^20^ suggested that, CCM produces a paradox in predicting the support pressure when it appears to decrease in relation to a more squeezing condition, whereas according to the 3D numerical modelling and the practical engineering, the support pressure is expected to increase remarkably as the rock mass becomes poorer and poorer in terms of geotechnical quality. They attributed the smaller support pressure estimated by CCM approaches to the underestimate of the stress relief associated with plastic yield of the ground ahead of the face. This is still unclear, so it is convenient to critically review the potential reasons behind this CCM outputs. Furthermore, Zhao et al.^17^, Cantieni and Anagnostou^18,19^, Alejano^12^ and Fuente et al.^20^ typically limited their discussion on this issue to squeezing conditions in poor rock masses submitted to high stress fields, so it would be convenient to extend the discussion to different geological conditions in order to advance towards an in-depth understanding of CCM and the further application of the CCM in practical tunnelling engineering.

In this paper, some of the mentioned limitations of CCM associated to an inappropriate representation of three-dimensionality are investigated by comparing the results obtained by the CCM and 3D numerical modelling. The procedure for solving the interaction between the rock mass and liner by the CCM is proposed. The rock deformation, support pressure and liner stress as obtained according to CCM and 3D numerical modelling under multiple geological conditions are compared. Based on the comparison of these results, some relevant reasons why CCM results in different rock deformation and support pressure in comparing to the 3D numerical modelling are elaborated. Some guidelines are eventually given regarding support design in the application of CCM and 3D numerical modelling.

## Problem description

### Basic assumptions

**Figure 1:**
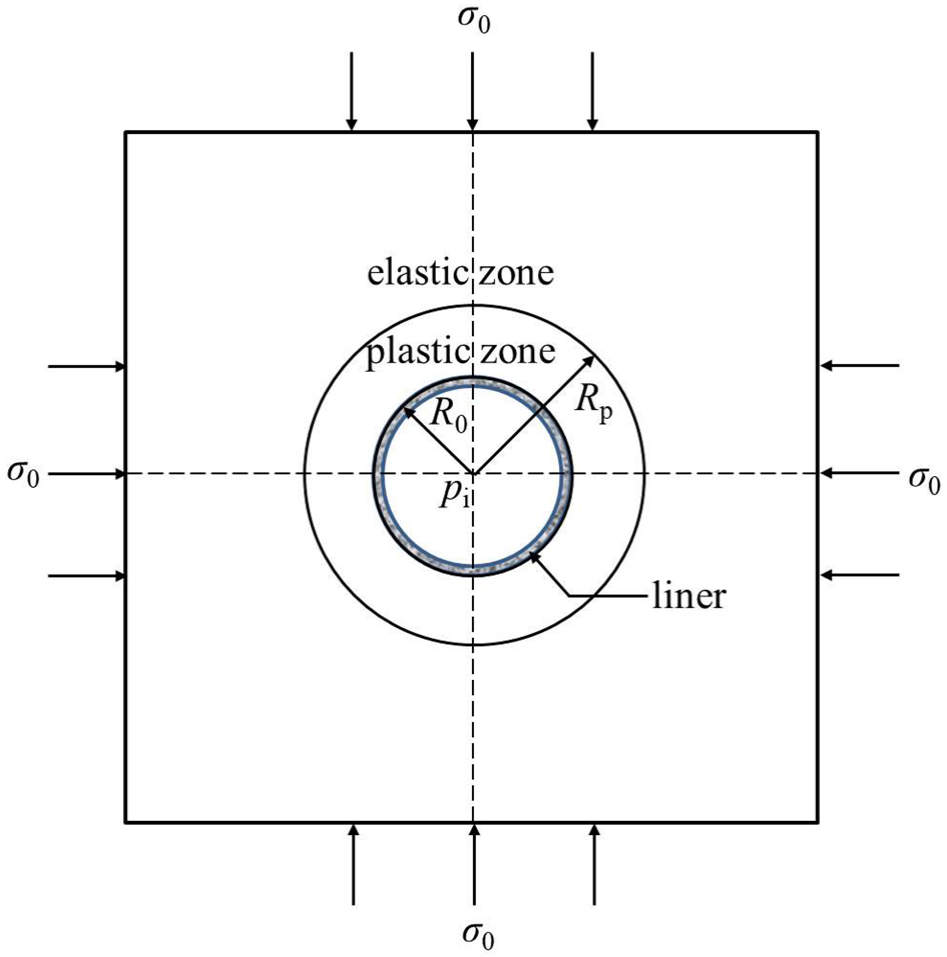
Schematic graph of rock mass, tunnel and liner.

As shown in Fig. 1, the following assumptions are to be adopted in this study:

#### Rock mass behaviour

The rock is isotropic, continuous, finite and initially elastic. In the vicinity of the tunnel face during the tunnel advance, the rock mass is gradually deformed and unloaded. The deformed rock mass is assumed to behave elastically or elastic-perfectly plastically.

#### Tunnels

As the CCM is discussed in this study, the complex sequence excavation method is not considered herein; instead, the full-face excavation method is assumed. The opening is circular with a radius of *R*_0_ and with a hydrostatic stress field *σ*_0_. In the plane perpendicular to the axial axis of the tunnel, a plane strain condition is postulated.

#### Support features

The purpose of this study is to analyse the difference between the CCM and 3D numerical modelling results. A simple liner support is considered, avoiding complex reinforcement such as bolts or forepole umbrellas. Further, the liner is assumed to be installed immediately after each excavation step.

### Convergence-confinement method (CCM)

**Figure 2:**
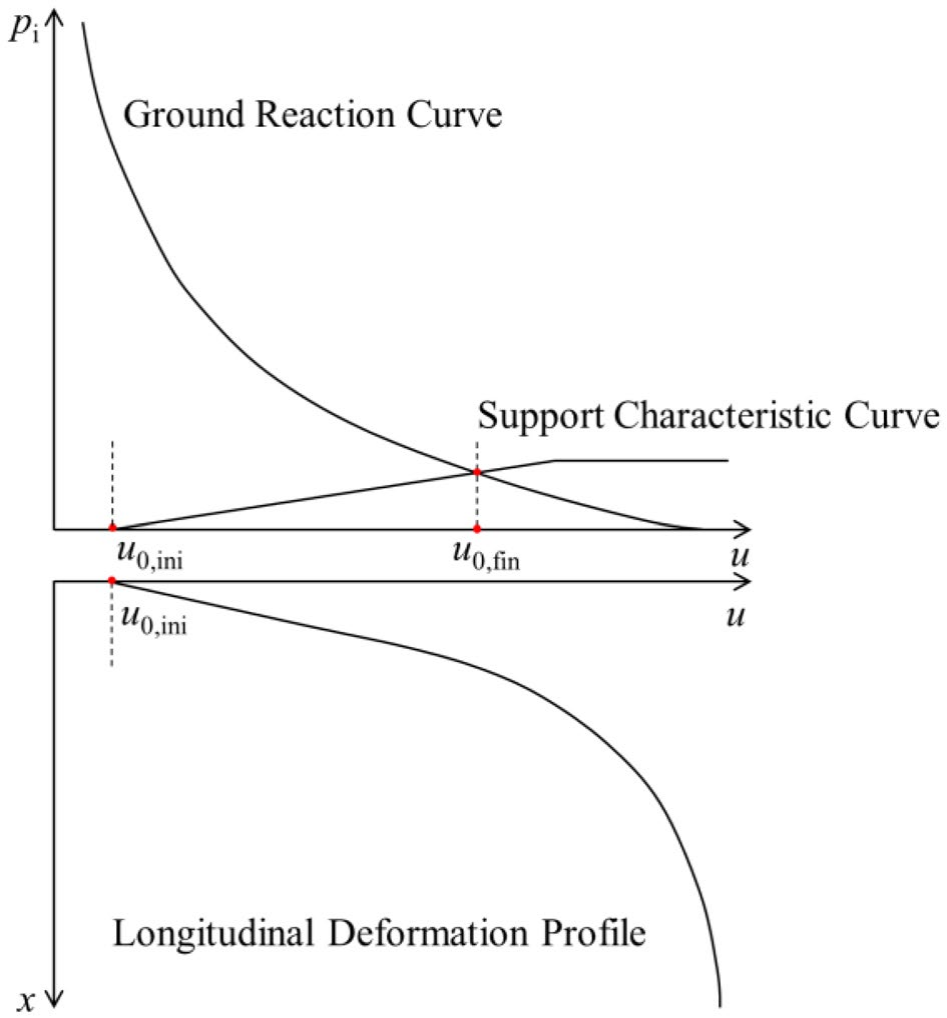
Schematic graph of CCM.

As shown in Fig. 2, the CCM is a tool to estimate the support loads during tunnelling through presenting the interplay between the rock mass and the installed support. The three basic components of the convergence-confinement method are: GRC, LDP and SCC.

The GRC is a characteristic line that records the decrease of a fictitious support pressure, which reflects the tunnel excavation process as the tunnel is being excavated past the section of interest. The LDP is a graphical representation of the radial rock deformation that occurs along the axial axis of an unsupported tunnel for the sections located ahead of and behind the face. The SCC is defined as the relationship between the increase of rock deformation and the decrease of internal pressure on the rock mass. The internal pressure is on the interface between the rock mass and liner.

The interpretation of the interaction among the LDP, GRC and SCC allows to define the internal pressure that the rock mass transmits to the liner as the face advances. The LDP defines the initial point of SCC at the abscissa of the GRC. In the LDP curve, *x* is the distance from the tunnel face along the tunnel direction. At the tunnel face (*x* = 0), the rock mass has already experienced certain amount of deformation, i.e., the initial rock deformation *u*_0,ini_. It should be noted that *u*_0,ini_ is variable: it is fairly small as the liner is installed early, and it becomes large as the support is installed late. In this study, *u*_0,ini_ is assumed as the rock deformation of the tunnel periphery at the tunnel face.

As the tunnel face advances, the stress to which the unexcavated rock mass was submitted, is continuously relieved. Due to this stress relief, the rock mass squeezes the liner. As a result, the internal pressure on the liner and the radial deformation of the liner are increased in SCC. As long as the tunnel face advances far from the analysed section, the system of the rock-liner reaches equilibrium. As shown in Fig. 2, the intersection point between SCC and GRC marks the equilibrium of deformation for a final value *u*_0,fin_ and at a typically low level of internal pressure.

### Understanding of 3D tunnelling effect

The 3D tunnelling effect during excavation can be well reflected by 3D numerical modelling, whereas it cannot be fully represented by CCM. In this study, the 3D tunnelling effect is believed to be composed of the group effect of liners and the disturbance effect of the tunnel face. These effects are thought to be the key factors that give rise to the different results of the two methods, as explained below.

#### Group effect of liners

**Figure 3:**
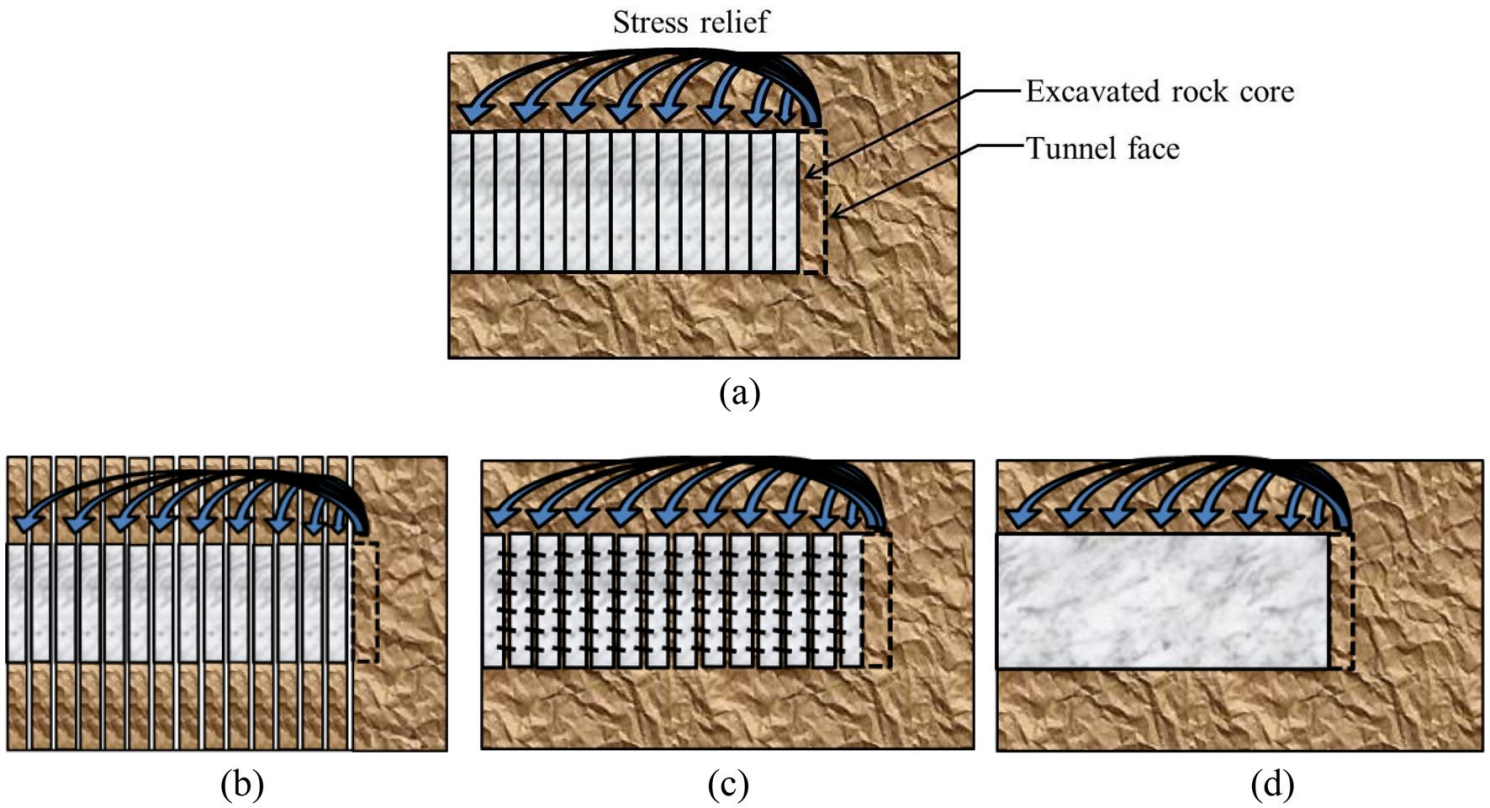
Schematic graph of (**a**) stress relief during tunnelling advance; (**b**) connections of adjacent liners by CCM; (**c**) connection of adjacent liners by TBMs; (**d**) connection of adjacent liners by drill and blast method.

Figure 3a plots that, as the rock mass at the tunnel face is excavated with an excavation step, the stresses in the excavated rock core are relieved and are transmitted to the liners. As shown in Fig. 3b, based on the CCM, the liners within each section suffer from the stress relief independently. In other words, CCM regards the connections of the adjacent liners to be free when solving out the internal support pressure and rock deformation at the location of interest. According to this method, during the tunnelling advance, the rock-liner interaction at the location of interest has no effect on other sections along the longitudinal direction.

However, in practical tunnel engineering or according to 3D numerical modelling, as far as the tunnel advances in a more or less continuous way, the liner at the location of interest is inevitably influenced by the adjacent ones. For example, as shown in Fig. 3c, for TBMs, the adjacent segmental liners are commonly connected through the joints and bolts. As shown in Fig. 3d, for the drill and blast method, the liners, in case that they consist of concrete and steel sets, are installed step by step. This means that the liners are connected in the longitudinal direction with different rigidity or stiffness. Hence, in the application of CCM, it is not rigorous to neglect the connections of the adjacent liners in the longitudinal direction during tunnelling advance.

**Figure 4:**
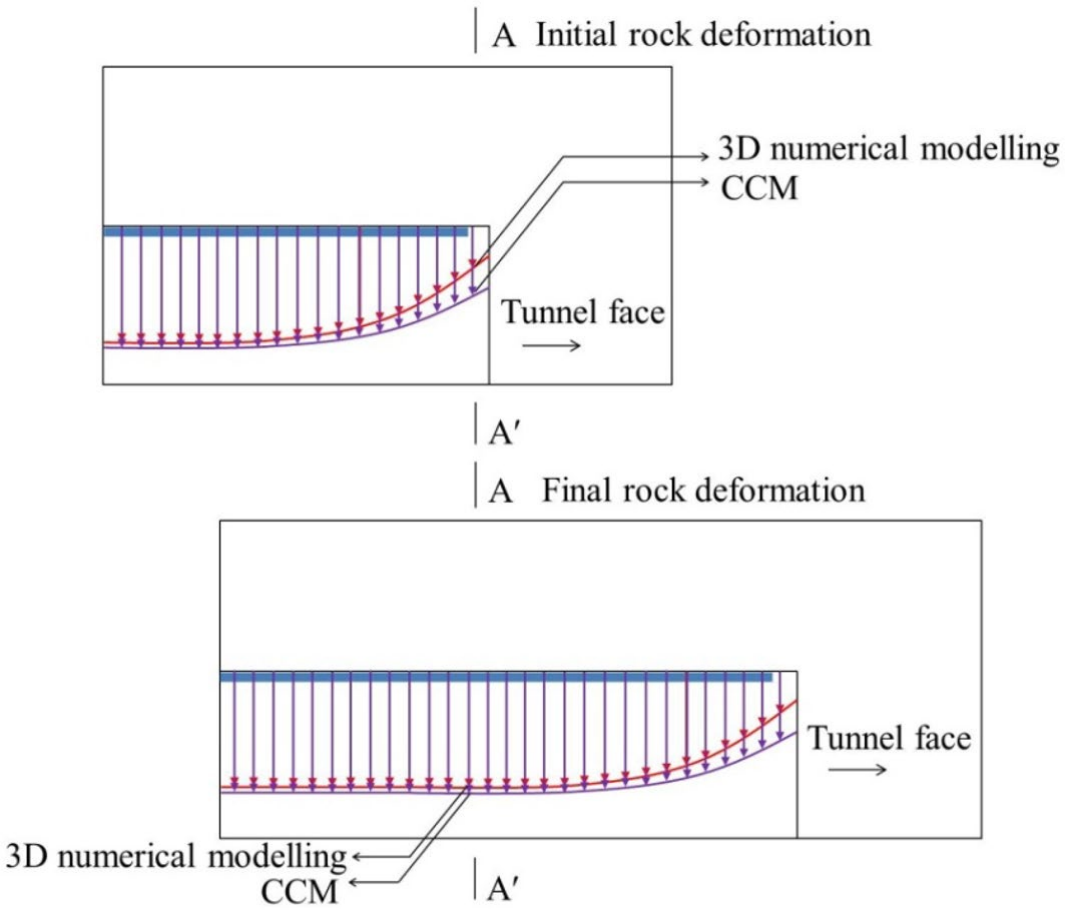
Schematic graph of positions of initial rock deformation and final rock deformation by 3D numerical modelling and CCM.

Herein, the consideration of the connection of adjacent liners is defined as the group effect of liners. As plotted in Fig. 4, at the initial state, because of the existence of the group liners, the initial rock deformation by 3D numerical modelling is relatively small, whereas that by the CCM is large since it is obtained through LDP, which have not considered the liner installation.

For the 3D numerical modelling, due to the constraint of the adjacent liners, the increment of the rock deformation at each excavation step is low, which leads to a small final rock deformation (herein, an excavation step denotes longitudinal length of drilling the tunnel). However, it is shown in Fig. 4 that, as the liner at each excavation step is assumed to deform independently in CCM, the final rock deformation derived from the CCM approach tends to be larger than that observed in 3D models.

#### Disturbance effect of tunnel face

The disturbance effect of the tunnel face is a reflection of the stress relief due to excavations. This effect renders the rock deformation in the 3D numerical modelling higher than CCM, which is opposite to the group effect of the liners.

**Figure 5:**
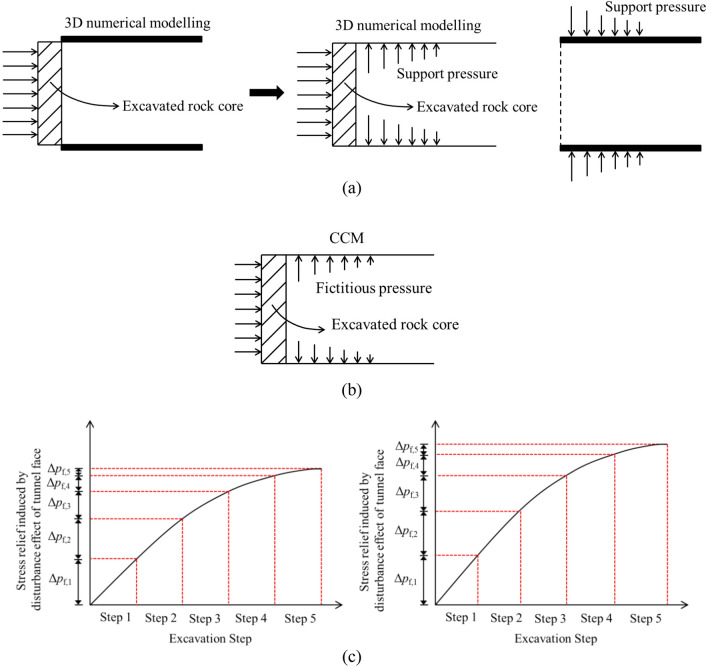
Schematic graph of stress relief at the tunnel face (**a**) for 3D numerical modelling (**b**) for CCM (**c**) comparison by 3D numerical modelling and CCM for different excavation steps.

**Figure 6:**
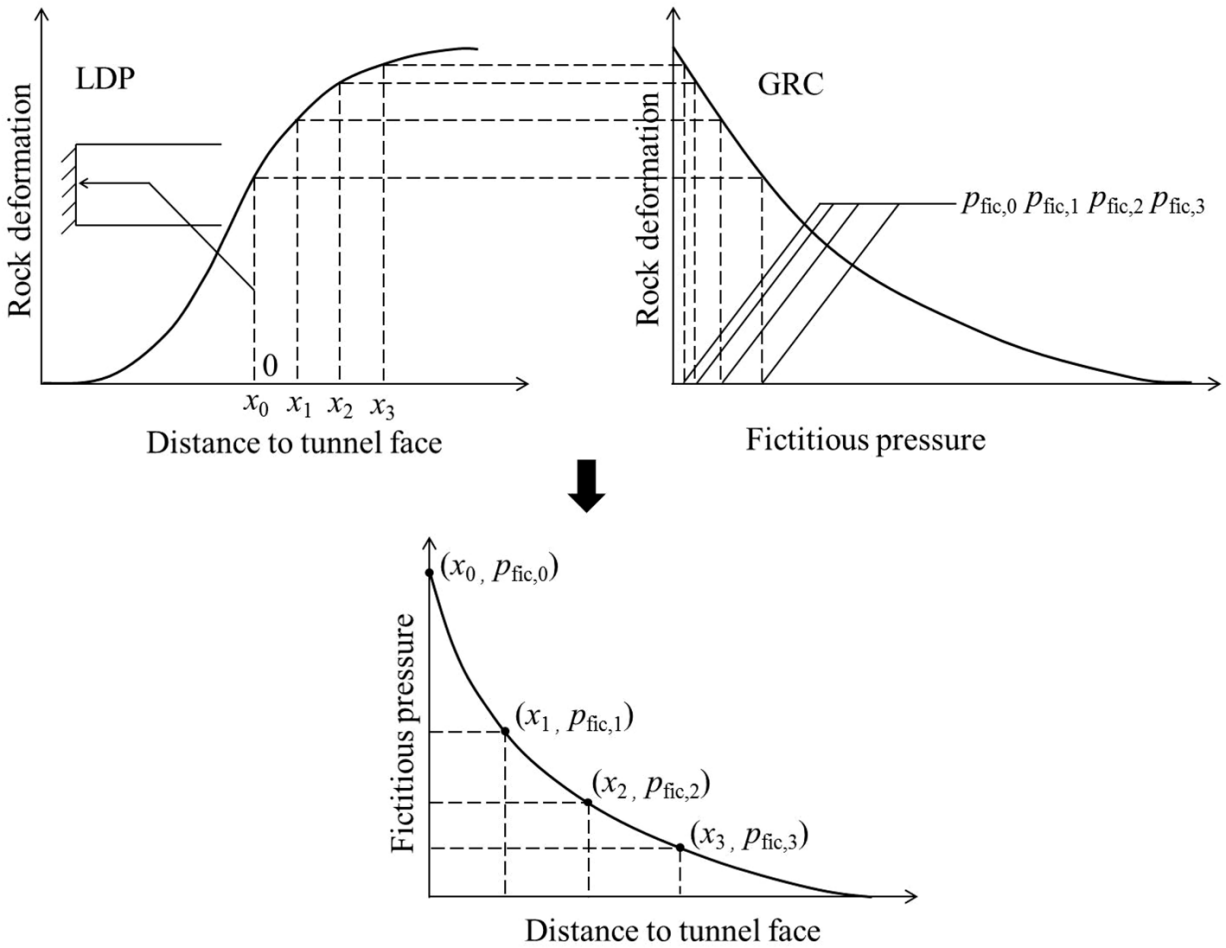
Schematic graph of the method to obtain fictitious pressure along the longitudinal direction^20^.

As plotted in Fig. 5, as for CCM, because LDP is solved out without the liners, the disturbance effect of the tunnel face is induced by the stress relief through excavating the rock core without reactions to the liner at each excavation step. Based on CCM, the solution of the stress relief can be referred to the research proposed by Cui et al.^21^. Figure 6 indicates that the fictitious pressure represents a self-support capacity of the rock mass in the vicinity of the tunnel face, which can be calculated by coupling the GRC and LDP. Actually, there is a relation between the fictitious pressure and the stress relief, i.e.:

1 $$\left( {{{p_{fic} } \mathord{\left/ {\vphantom {{p_{fic} } {\sigma_{0} }}} \right. \kern-0pt} {\sigma_{0} }}} \right)\% + \left( {f_{0} } \right)\% = 100\%$$

where *f*_0_ is the stress relief, *p*_fic_ is the fictitious pressure, *σ*_0_ is the initial stress. An excavation step can be the abscissa of the LDP or the stress relief or the fictitious pressure.

However, as plotted in Fig. 5, in the 3D numerical modelling, as the liner provides the support pressure to the rock mass at the tunnel periphery, on one hand, it constrains the rock deformation at the tunnel periphery, which correlates to the group effect of liners that is introduced above; but on the other hand, it prompts the longitudinal deformation to increase at the tunnel face as the force transfers to the tunnel face, this brings more stress relief at the tunnel face. Hence, due to the installation of the liners, the 3D numerical modelling is encountered with more stress relief at each excavation step.

It should be noticed that the disturbance effect of the tunnel face and the group effect of liners take place simultaneously during the 3D excavation process. The two effects are opposite. The magnitudes of the two effects depend on several factors such as the geological condition, the strength and deformability of the rock mass and the support stiffness, which results in differences between the 3D numerical modelling and CCM. In the following, the occurrence of the two effects will be illustrated by comparing 3D numerical modelling and CCM results.

## Solutions of CCM and 3D numerical modelling

### Numerical solution of CCM

#### GRC

The GRC solutions used herein is a special case of the general formulation presented in Cui et al.^22^ for strain-softening problems. However, for the sake of clarity, a brief description is presented here. More detailed comments on this approach can be found in the mentioned reference.

**Figure 7:**
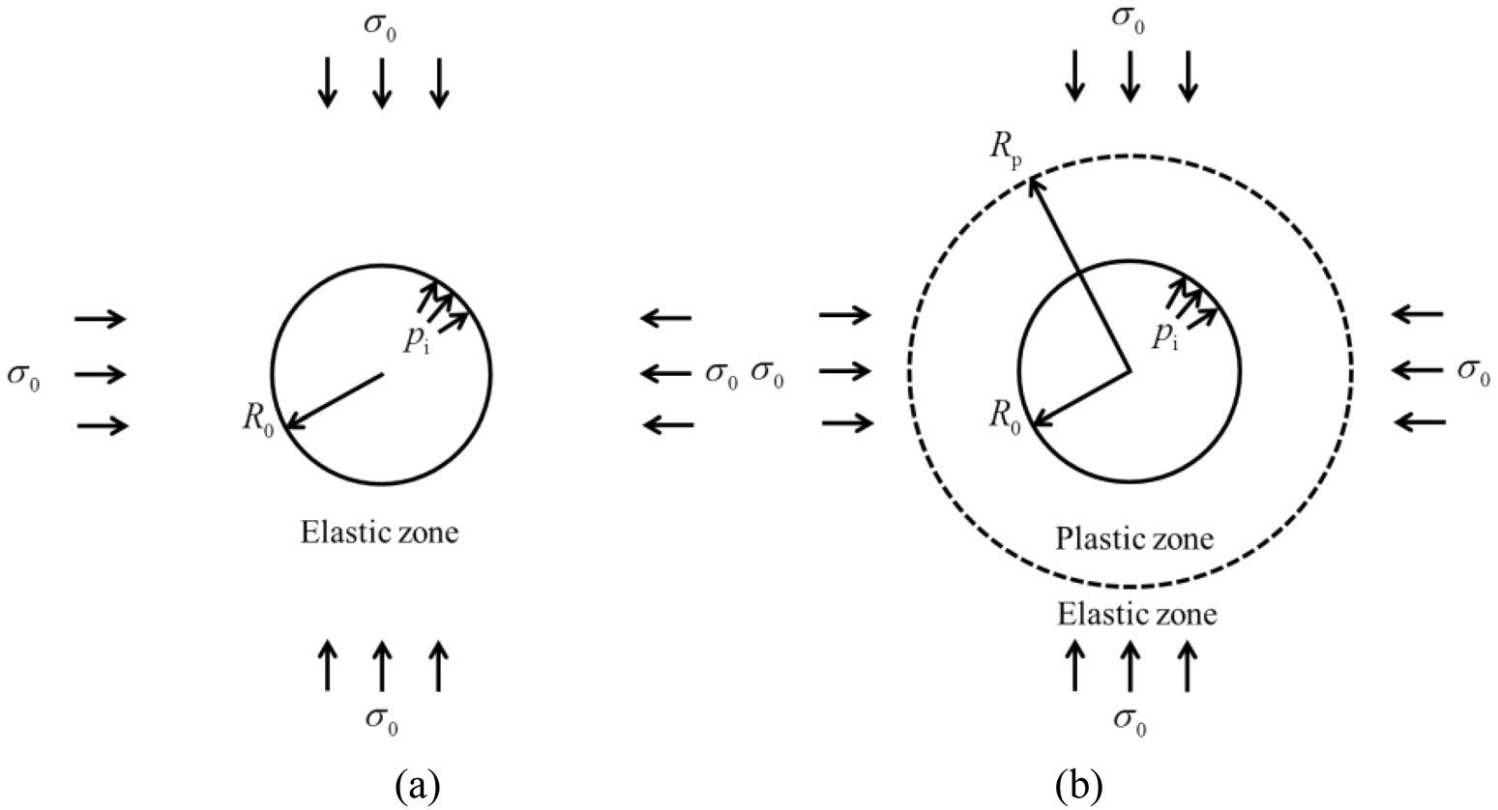
Excavation model for solution of GRC (**a**) rock mass is composed of the elastic zone (**b**) rock mass is composed of the elastic and plastic zones.

The basic excavation model is plotted in Fig. 7. The governing equilibrium equations for the stress components *σ*_r_ and *σ*_θ_, and the strain components *ε*_r_ and *ε*_θ_, are presented as follows:

2 $$\frac{{d\sigma_{{\text{r}}} }}{r} + \frac{{\sigma_{{\text{r}}} - \sigma_{{\uptheta }} }}{r} = 0$$

3 $$\varepsilon_{{\text{r}}} = \frac{du}{{dr}},\varepsilon_{{\uptheta }} = \frac{u}{r}$$

where *u* denotes the rock displacement in the radial direction, and *r* denotes the distance within the rock mass from the center of the circular opening.

The rock mass is assumed to obey the generalised Hoek–Brown (H-B) failure criterion. Since *σ*_r_ and *σ*_θ_ are equal to *σ*_3_ and *σ*_1_, the failure criterion can be formulated as^23^:

4 $$f\left( {\sigma_{{\uptheta }} ,\sigma_{{\text{r}}} } \right) = \sigma_{{\uptheta }} - \sigma_{{\text{r}}} - \sigma_{{{\text{ci}}}} \left( {{{m_{{\text{b}}} \sigma_{{\text{r}}} } \mathord{\left/ {\vphantom {{m_{{\text{b}}} \sigma_{{\text{r}}} } {\sigma_{{{\text{ci}}}} + s}}} \right. \kern-0pt} {\sigma_{{{\text{ci}}}} + s}}} \right)^{a}$$

where *m*_b_, *s*, *a* are the H–B strength constants, *σ*_ci_ is the uniaxial compressive strength of intact rock.

The radial stress at the elasto-plastic boundary, *σ*_r2_, can be obtained using the following equation by Newton–Raphson method,

5 $$\sigma_{{{\text{ci}}}} \left( {m_{{\text{b}}} {{\sigma_{{{\text{r2}}}} } \mathord{\left/ {\vphantom {{\sigma_{{{\text{r2}}}} } {\sigma_{{{\text{ci}}}} }}} \right. \kern-0pt} {\sigma_{{{\text{ci}}}} }} + s} \right)^{a} + 2\sigma_{{{\text{r2}}}} - 2\sigma_{0} = 0$$

The radius of plastic zone *R*_p_ can be obtained as^21^,

6 $$R_{{\text{p}}} = R_{0} \exp \left[ {\frac{{\left( {m_{{\text{b}}} {{\sigma_{{{\text{r2}}}} } \mathord{\left/ {\vphantom {{\sigma_{{{\text{r2}}}} } {\sigma_{{{\text{ci}}}} }}} \right. \kern-0pt} {\sigma_{{{\text{ci}}}} }} + s} \right)^{a} - \left( {{{\sigma_{{{\text{r2}}}} } \mathord{\left/ {\vphantom {{\sigma_{{{\text{r2}}}} } {\sigma_{{{\text{ci}}}} }}} \right. \kern-0pt} {\sigma_{{{\text{ci}}}} }} + s} \right)^{a} }}{{m_{{\text{b}}} \left( {1 - a} \right)}}} \right]$$

As shown in Fig. 7a, if *p*_i_ exceeds *σ*_r2_, the rock mass is in elastic state and therefore the rock deformation at the tunnel periphery *u*_0_ can be written as^24^:

7 $$u_{0} = {{\left( {1 + \mu } \right)\left( {\sigma_{0} - p_{{\text{i}}} } \right)R_{0} } \mathord{\left/ {\vphantom {{\left( {1 + \mu } \right)\left( {\sigma_{0} - p_{{\text{i}}} } \right)R_{0} } {E_{{\text{r}}} }}} \right. \kern-0pt} {E_{{\text{r}}} }}$$

As plotted in Fig. 7b, if *p*_i_ is less than *σ*_r2_, the rock mass presents an elastic zone and a plastic zone. Due to the nonlinear characteristic of the H-B failure criterion, the solution should resort to the numerical method. In this study, the plastic zone is divided into a set of concentric annuli, where *r*_(*i*)_ and *r*_(*i*-1)_ are the radii of the inner and outer boundaries of the *i*th annulus. *i* = 0 and *n* correspond to the locations at the elasto-plastic boundary and at the tunnel periphery, respectively.

A constant radial stress increment Δ*σ*_r_ is assumed for each annulus, i.e.:

8 $$\Delta \sigma_{{\text{r}}} = \sigma_{{{\text{r}}\left( i \right)}} - \sigma_{{{\text{r}}\left( {i - 1} \right)}} = {{\left( {p_{{\text{i}}} - \sigma_{{{\text{r2}}}} } \right)} \mathord{\left/ {\vphantom {{\left( {p_{{\text{i}}} - \sigma_{{{\text{r2}}}} } \right)} n}} \right. \kern-0pt} n}$$

where *n* is the total number of the concentric annuli; and *σ*r(*i*) is the radial stress at *r* = *r*_(*i*)_ (*i* = 1, 2, …, *n*).

According to the Cui et al.’s solution^25^, the tangential strain ɛ_θ(i)_ at *r* = *r*_(i)_ can be solved as:

9 $$\varepsilon_{{{\uptheta }\left( i \right)}} = {{\left[ {A_{(i - 1)} \left( {{{r_{\left( i \right)} } \mathord{\left/ {\vphantom {{r_{\left( i \right)} } {r_{{\left( {i - 1} \right)}} }}} \right. \kern-0pt} {r_{{\left( {i - 1} \right)}} }} - 1} \right) + \varepsilon_{{{\uptheta }\left( {i - 1} \right)}} } \right]} \mathord{\left/ {\vphantom {{\left[ {A_{(i - 1)} \left( {{{r_{\left( i \right)} } \mathord{\left/ {\vphantom {{r_{\left( i \right)} } {r_{{\left( {i - 1} \right)}} }}} \right. \kern-0pt} {r_{{\left( {i - 1} \right)}} }} - 1} \right) + \varepsilon_{{{\uptheta }\left( {i - 1} \right)}} } \right]} {\left[ {{{r_{\left( i \right)} } \mathord{\left/ {\vphantom {{r_{\left( i \right)} } {r_{{\left( {i - 1} \right)}} }}} \right. \kern-0pt} {r_{{\left( {i - 1} \right)}} }} + K_{{\uppsi }} \left( {{{r_{\left( i \right)} } \mathord{\left/ {\vphantom {{r_{\left( i \right)} } {r_{{\left( {i - 1} \right)}} }}} \right. \kern-0pt} {r_{{\left( {i - 1} \right)}} }} - 1} \right)} \right]}}} \right. \kern-0pt} {\left[ {{{r_{\left( i \right)} } \mathord{\left/ {\vphantom {{r_{\left( i \right)} } {r_{{\left( {i - 1} \right)}} }}} \right. \kern-0pt} {r_{{\left( {i - 1} \right)}} }} + K_{{\uppsi }} \left( {{{r_{\left( i \right)} } \mathord{\left/ {\vphantom {{r_{\left( i \right)} } {r_{{\left( {i - 1} \right)}} }}} \right. \kern-0pt} {r_{{\left( {i - 1} \right)}} }} - 1} \right)} \right]}}$$

10 $$\varepsilon_{{{\text{r}}\left( i \right)}} = - K_{{\uppsi }} \varepsilon_{{{\uptheta }\left( i \right)}} + {{A_{(i - 1)} \left( {{{r_{\left( i \right)} } \mathord{\left/ {\vphantom {{r_{\left( i \right)} } {r_{{\left( {i - 1} \right)}} - 1}}} \right. \kern-0pt} {r_{{\left( {i - 1} \right)}} - 1}}} \right)} \mathord{\left/ {\vphantom {{A_{(i - 1)} \left( {{{r_{\left( i \right)} } \mathord{\left/ {\vphantom {{r_{\left( i \right)} } {r_{{\left( {i - 1} \right)}} - 1}}} \right. \kern-0pt} {r_{{\left( {i - 1} \right)}} - 1}}} \right)} {\left[ {1 - {{r_{{\left( {i - 1} \right)}} } \mathord{\left/ {\vphantom {{r_{{\left( {i - 1} \right)}} } {r_{\left( i \right)} }}} \right. \kern-0pt} {r_{\left( i \right)} }}} \right]}}} \right. \kern-0pt} {\left[ {1 - {{r_{{\left( {i - 1} \right)}} } \mathord{\left/ {\vphantom {{r_{{\left( {i - 1} \right)}} } {r_{\left( i \right)} }}} \right. \kern-0pt} {r_{\left( i \right)} }}} \right]}}$$

The corresponding solutions for *r*_(i)_/*r*_(i-1), *A*(i-1)_, and other unknowns are presented in the Appendix A.

The rock deformation at *r* = *r*_(i)_ is solved as:

11 $$u_{r\left( i \right)} = r_{{\left( {i - 1} \right)}} {{\left[ {A_{{\left( {i - 1} \right)}} \left( {{{r_{\left( i \right)} } \mathord{\left/ {\vphantom {{r_{\left( i \right)} } {r_{{\left( {i - 1} \right)}} }}} \right. \kern-0pt} {r_{{\left( {i - 1} \right)}} }} - 1} \right) + {{u_{{r\left( {i - 1} \right)}} } \mathord{\left/ {\vphantom {{u_{{r\left( {i - 1} \right)}} } {r_{{\left( {i - 1} \right)}} }}} \right. \kern-0pt} {r_{{\left( {i - 1} \right)}} }}} \right]} \mathord{\left/ {\vphantom {{\left[ {A_{{\left( {i - 1} \right)}} \left( {{{r_{\left( i \right)} } \mathord{\left/ {\vphantom {{r_{\left( i \right)} } {r_{{\left( {i - 1} \right)}} }}} \right. \kern-0pt} {r_{{\left( {i - 1} \right)}} }} - 1} \right) + {{u_{{r\left( {i - 1} \right)}} } \mathord{\left/ {\vphantom {{u_{{r\left( {i - 1} \right)}} } {r_{{\left( {i - 1} \right)}} }}} \right. \kern-0pt} {r_{{\left( {i - 1} \right)}} }}} \right]} {\left[ {{{r_{\left( i \right)} } \mathord{\left/ {\vphantom {{r_{\left( i \right)} } {r_{{\left( {i - 1} \right)}} }}} \right. \kern-0pt} {r_{{\left( {i - 1} \right)}} }} + K_{{\uppsi }} \left( {{{r_{\left( i \right)} } \mathord{\left/ {\vphantom {{r_{\left( i \right)} } {r_{{\left( {i - 1} \right)}} }}} \right. \kern-0pt} {r_{{\left( {i - 1} \right)}} }} - 1} \right)} \right]}}} \right. \kern-0pt} {\left[ {{{r_{\left( i \right)} } \mathord{\left/ {\vphantom {{r_{\left( i \right)} } {r_{{\left( {i - 1} \right)}} }}} \right. \kern-0pt} {r_{{\left( {i - 1} \right)}} }} + K_{{\uppsi }} \left( {{{r_{\left( i \right)} } \mathord{\left/ {\vphantom {{r_{\left( i \right)} } {r_{{\left( {i - 1} \right)}} }}} \right. \kern-0pt} {r_{{\left( {i - 1} \right)}} }} - 1} \right)} \right]}}$$

Based on Eq. (11), the rock deformation at the tunnel periphery *u*_0_ can be obtained as,

12 $$u_{0} = u_{r\left( n \right)} = r_{{\left( {n - 1} \right)}} {{\left[ {A_{{\left( {n - 1} \right)}} \left( {{{r_{\left( n \right)} } \mathord{\left/ {\vphantom {{r_{\left( n \right)} } {r_{{\left( {n - 1} \right)}} }}} \right. \kern-0pt} {r_{{\left( {n - 1} \right)}} }} - 1} \right) + {{u_{{r\left( {n - 1} \right)}} } \mathord{\left/ {\vphantom {{u_{{r\left( {n - 1} \right)}} } {r_{{\left( {n - 1} \right)}} }}} \right. \kern-0pt} {r_{{\left( {n - 1} \right)}} }}} \right]} \mathord{\left/ {\vphantom {{\left[ {A_{{\left( {n - 1} \right)}} \left( {{{r_{\left( n \right)} } \mathord{\left/ {\vphantom {{r_{\left( n \right)} } {r_{{\left( {n - 1} \right)}} }}} \right. \kern-0pt} {r_{{\left( {n - 1} \right)}} }} - 1} \right) + {{u_{{r\left( {n - 1} \right)}} } \mathord{\left/ {\vphantom {{u_{{r\left( {n - 1} \right)}} } {r_{{\left( {n - 1} \right)}} }}} \right. \kern-0pt} {r_{{\left( {n - 1} \right)}} }}} \right]} {\left[ {{{r_{\left( n \right)} } \mathord{\left/ {\vphantom {{r_{\left( n \right)} } {r_{{\left( {n - 1} \right)}} }}} \right. \kern-0pt} {r_{{\left( {n - 1} \right)}} }} + K_{{\uppsi }} \left( {{{r_{\left( n \right)} } \mathord{\left/ {\vphantom {{r_{\left( n \right)} } {r_{{\left( {n - 1} \right)}} }}} \right. \kern-0pt} {r_{{\left( {n - 1} \right)}} }} - 1} \right)} \right]}}} \right. \kern-0pt} {\left[ {{{r_{\left( n \right)} } \mathord{\left/ {\vphantom {{r_{\left( n \right)} } {r_{{\left( {n - 1} \right)}} }}} \right. \kern-0pt} {r_{{\left( {n - 1} \right)}} }} + K_{{\uppsi }} \left( {{{r_{\left( n \right)} } \mathord{\left/ {\vphantom {{r_{\left( n \right)} } {r_{{\left( {n - 1} \right)}} }}} \right. \kern-0pt} {r_{{\left( {n - 1} \right)}} }} - 1} \right)} \right]}}$$

It is noted that Eq. (12) should be obtained step by step from *i* = 1 to *i* = *n*.

#### LDP

The equation for LDP proposed by Vlachopoulos and Diederichs^26^ correlates the rock deformation near the tunnel face to the extent of the plastic zone. Initially proposed to elastic perfectly-plastic materials, Alejano et al.^13^ showed it can be extended to strain-softening rock masses. Owing to its convenience, this equation for LDP (i.e., V-D equation) is widely adopted.

However, V-D equation is solved out based on a 2D plane axisymmetric finite element numerical modelling, in fact, the 3D numerical modelling is more appropriate. Moreover, in the original research^26^, merely twelve analysis cases were presented to predict rock deformation, which seems to be rather limited. To overcome the limitations, Shen et al.^27,28^ proposed a modified fitting equation of LDP based on a large number of cases analysed by means of 3D numerical models.

13 $$u^{*} = \exp \left( \begin{gathered} a_{1} + b_{1} \times X^{*} + b_{2} \times R^{*} + \hfill \\ c_{1} \times (X^{*} )^{2} + c_{2} \times X^{*} \times R^{*} + c_{3} \times (R^{*} )^{2} + \hfill \\ d_{1} \times (X^{*} )^{3} + d_{2} \times (X^{*} )^{2} \times R^{*} + d_{3} \times X^{*} \times (R^{*} )^{2} + d_{4} \times (R^{*} )^{3} + \hfill \\ e_{1} \times (X^{*} )^{4} + e_{2} \times (X^{*} )^{3} \times R^{*} + e_{3} \times (X^{*} )^{2} \times (R^{*} )^{2} + e_{4} \times X^{*} \times (R^{*} )^{3} + e_{5} \times (R^{*} )^{4} \hfill \\ \end{gathered} \right), \, X^{*} > 0$$

The coefficients in the equation include *a*_1_, *b*_1_, *b*_2_, *c*_1_*, c*_2_*, c*_3_*, d*_1_*, d*_2_*, d*_3_*, d*_4_*, e*_1_*, e*_2_*, e*_3_*, e*_4_*, e*_5_*.* The values of the coefficients are presented in Appendix B. The variables u^*^, X^*^ and R^*^ as shown in Eq. (13) are the dimensionless forms of initial rock deformation at the tunnel periphery *u*_0,ini_, the distance to the tunnel face *x*, and the radius of the plastic zone *R*_p_, according to the following equations,

14 $$u^{*} = \frac{{u_{0,ini} }}{{u_{0,\max } }},\;X^{*} = \frac{x}{{R_{0} }},\;R^{*} = \frac{{R_{p} }}{{R_{0} }}$$

#### SCC

The relation between the final rock deformation *u*_0,fin_ and final support pressure *p*_s,fin_ is written as:

15 $$K_{{\text{s}}} \left( {u_{{\text{0,fin}}} - u_{{\text{0,ini}}} } \right) = p_{{\text{s,fin}}}$$

where *u*_0,ini_ is the initial rock deformation. *K*_s_ in Eq. (15) is the stiffness of the liner, derived as^3^:

16 $$K_{{\text{s}}} = \frac{{E_{{\text{s}}} }}{{1 + \mu_{{\text{s}}} }} \cdot \frac{{\left[ {R_{0}^{2} - \left( {R_{0} - t_{{\text{s}}} } \right)^{2} } \right]}}{{\left[ {\left( {1 - 2\mu_{{\text{s}}} } \right)R_{0}^{2} + \left( {R_{0} - t_{{\text{s}}} } \right)^{2} } \right]}} \cdot \frac{1}{{R_{0} }}$$

According to Cui et al.’s study^22,25^, Eq. (15) can be rewritten as,

17 $$K_{{\text{s}}} \left( {\varepsilon_{{{\uptheta }\left( i \right)}} R_{0} - u_{{\text{0,ini}}} } \right) = \sigma_{{{\text{r}}\left( i \right)}}$$

18 $$\varepsilon_{{{\uptheta }\left( i \right)}} R_{0} { = }u_{{\text{0,fin}}}$$

19 $$\sigma_{{{\text{r}}\left( i \right)}} = p_{{\text{s,fin}}}$$

where *ε*_θ(*i*)_ and *σ*_r(*i*)_ in Eqs. (17–19) correspond to the strain and stress at a specific annulus; it should be noticed that *ε*_θ(*i*)_ and *σ*_r(*i*)_ in Eqs. (17–19) can be obtained when the full distributions of strain and stress are known, obtained according to Eqs. (9) and (10).where *E*_s_ is the elastic modulus of the liner, *μ*_s_ is Poisson’s ratio of the liner and *t*_s_ is the thickness of the liner. Remark that Eq. (16) is a representation of the SCC.

#### Procedure for solving out rock deformations and support pressures

**Figure 8:**
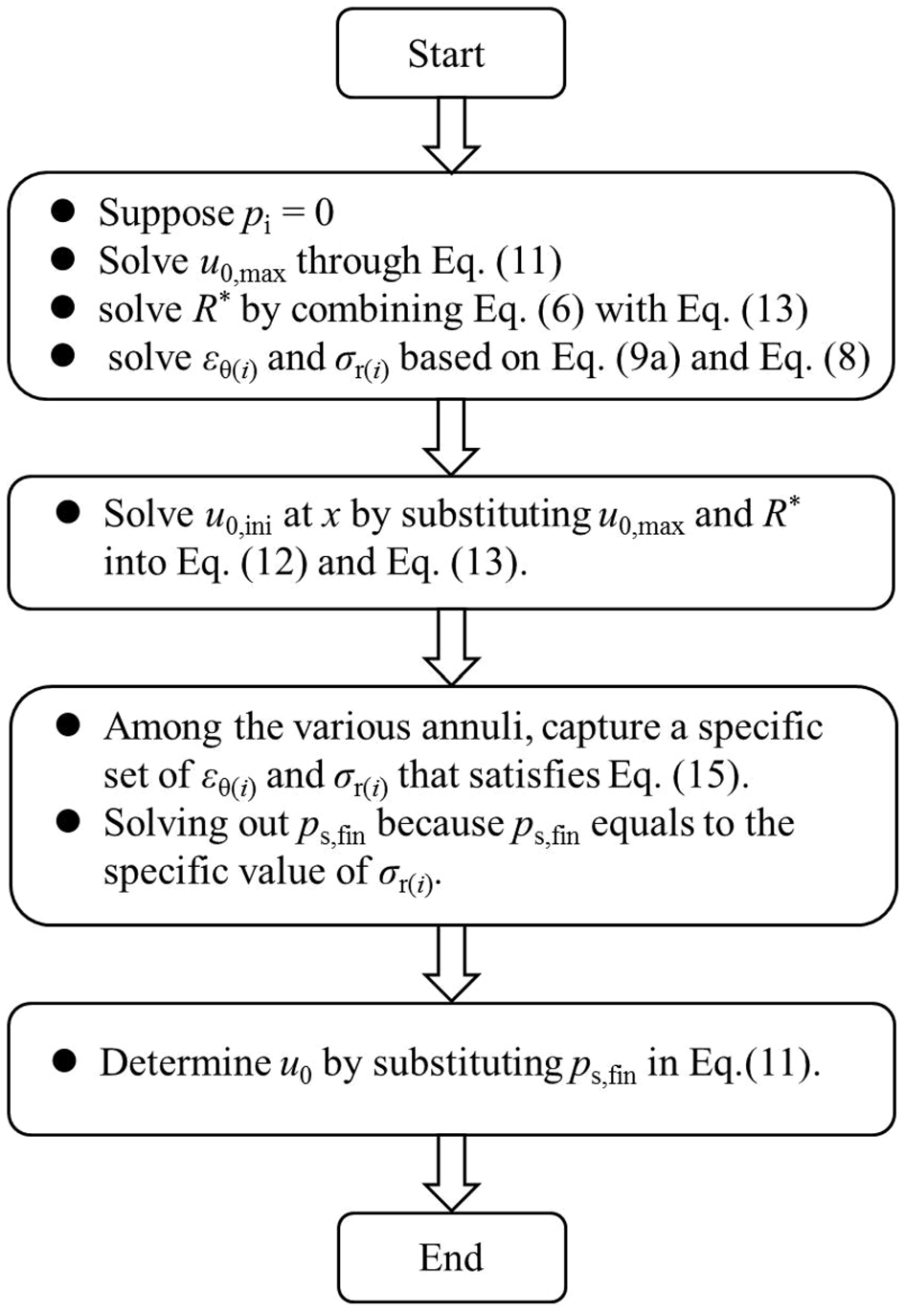
Flowchart of procedure for solving out rock deformation and support pressure.

The flowchart of the procedure is shown in Fig. 8. The procedure is summarised as follows,

1. Suppose *p*_i_ = 0, solve out *u*_0,max_, R^*^, *ε*_θ(*i*)_ and *σ*_r(*i*)_ at each annulus; specifically, solve out *u*_0,max_ through Eq. (12), solve out R^*^ by combining Eq. (6) with Eq. (14), solve out *ε*_θ(*i*)_ and *σ*_r(*i*)_ based on Eqs. (9) and (8).
2. Solve out *u*_0,ini_ at* x* by substituting *u*_0,max_ and R^*^ into Eqs. (13) and (14).
3. Among the various annuli, capture a specific set of *ε*_θ(*i*)_ and *σ*_r(*i*)_ that satisfies Eq. (16), solve out *p*_s,fin_ because *p*_s,fin_ equals to the specific value of *σ*_r(*i*)_ according to Eq. (19).
4. Determine *u*_0_ by substituting *p*_s,fin_ into Eq. (12).

### Solution of 3D Numerical modelling

**Figure 9:**
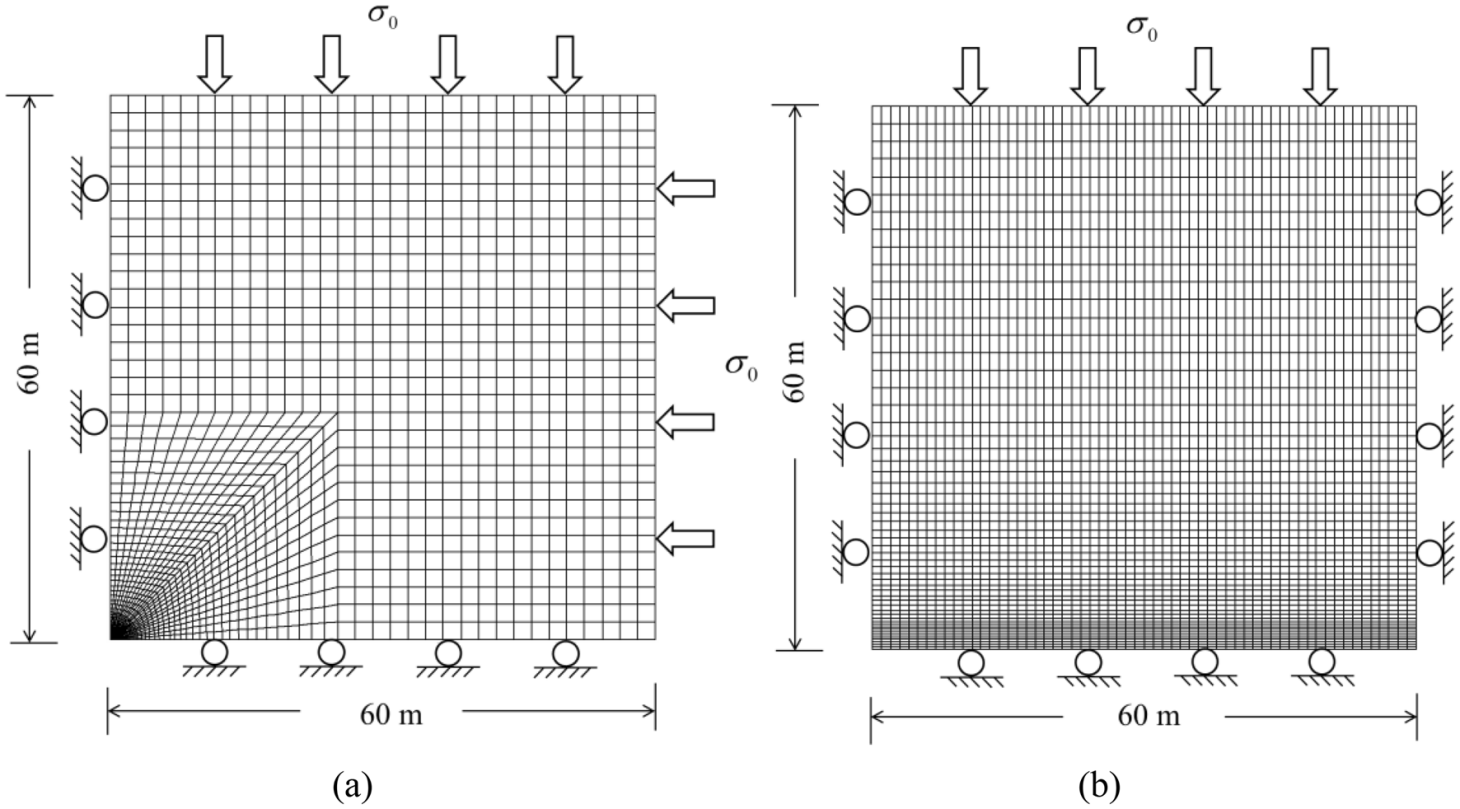
3D numerical modelling of simulation (**a**) in transverse section; (**b**) in longitudinal section.

A parametric analysis was performed with finite difference software FLAC3D 3.0^29^. The geometry of the model is shown in Fig. 9. To reduce the simulation time, only a quarter of the tunnel was modelled, which was considered adequate due to the symmetry. The dimensions of the model were 60 m in the horizontal, vertical and longitudinal directions. In the transverse section of the model, the mesh size was gradually reduced towards the tunnel zone. As shown in Fig. 9, the normal displacements were fixed at zero for all the boundaries except the upper surface boundary and right surface boundary. Homogeneous initial normal stresses *σ*_0_ were applied at the top and right boundaries.

A 3 m diameter circular tunnel was excavated and the full face excavation method was adopted. The rock mass was assumed to behave in an elastic-perfectly-plastic manner, obeying a H-B failure criterion. Non-associated flow rule was adopted by regarding the dilatancy angle *ψ* as 0. The liners were simulated using built-in shell elements that were uniformly distributed around the tunnel periphery. To represent CCM assumptions, the stiffness between the shell and the rock element was set to be infinitely large. The excavation step per cycle was simulated to 1 m. In the model, 35 excavation rounds were performed, and the excavation was then stopped at the 35th round.

Depending on whether the liner was installed or not, two analysis conditions were simulated. For the first condition, the modelling process at each excavation step included deleting the rock core and running to equilibrium. For the second condition, after deleting the rock core and running to equilibrium, the process included installing the shell element and running to equilibrium. The models for both cases were stopped after 35th rounds.

**Figure 10:**
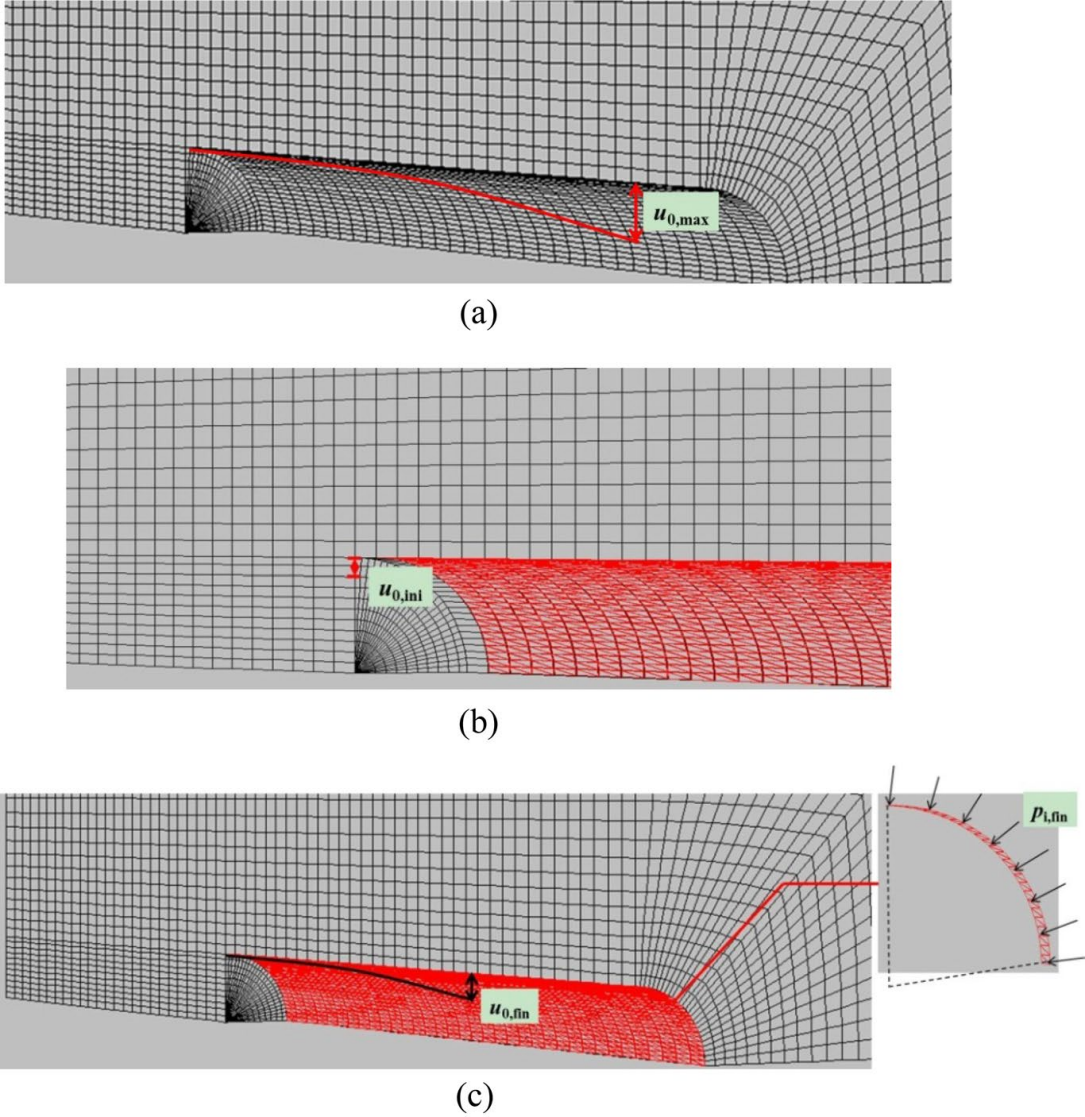
Rock deformations and support pressure according to 3D numerical modelling (**a**) maximum rock deformation (**b**) initial rock deformation (**c**) final rock deformation and final support pressure.

To compare with the solutions of CCM, different typical rock deformations or support pressure, for example, the maximum rock deformation *u*_0,max_, the initial rock deformation *u*_0,ini_, the final rock deformations *u*_0,fin_, and the final support pressure *p*_s,fin_ were obtained from the modelling. *u*_0,max_ was captured from the above described first analysis condition, but *u*_0,ini_, *u*_0,fin_ and *p*_s,fin_ were recovered from the second analysis condition. As plotted in Fig. 10, *u*_0,max_ is the maximum radial deformation of the liner along the longitudinal direction. *u*_0,fin_ and *p*_s,fin_ are respectively the maximum radial deformation and the maximum support pressure in the liner along the longitudinal direction, respectively. *u*_0,ini_ is the radial deformation at the tunnel face with the liner when the liner near the tunnel face is not yet installed.

## Result and discussion

Seven cases considering rock mass with different GSIs from Cui et al.’s study^24^ are presented for comparative purposes in Table 1. As GSI increases from 20 to 50, the rock mass quality improves. *σ*_0_ is assumed to range from 5 to 25 MPa with an interval of 5 MPa. Two types of the liners are presented in Table 2. One type is a rigid one and the other a flexible one. Combining 7 possible rock mass qualities, 5 initial stress levels and the 2 liner types, the authors have analysed 70 cases within this parametric analysis. The rock deformation and support pressure obtained by means of both the CCM and the 3D numerical modelling for the 70 cases are investigated in this section.

**Table 1:** Mechanical parameters of rock mass with different GSIs Cui et al.^24^.

GSI	*σ*_ci_/MPa	*m*_b_	*s*	*a*	*E*_r_/MPa	*μ*
50	95.274	2.405	0.003866	0.506	10614	0.25
45	82.749	1.857	0.002218	0.508	7583	0.25
40	70.652	1.421	0.001273	0.511	5336	0.25
35	59.282	1.074	0.00073	0.516	3691	0.25
30	48.894	0.799	0.000419	0.522	2502	0.25
25	39.674	0.582	0.00024	0.531	1661	0.25
20	31.711	0.411	0.000138	0.544	1083	0.25

**Table 2:** Mechanical parameters of liners.

	*t*_s_/m	*E*_s_/GPa	*u*_s_
Rigid liner	0.21	30	0.15
Flexible liner	0.18	20	0.15

### Comparison of maximum rock deformation

**Figure 11:**
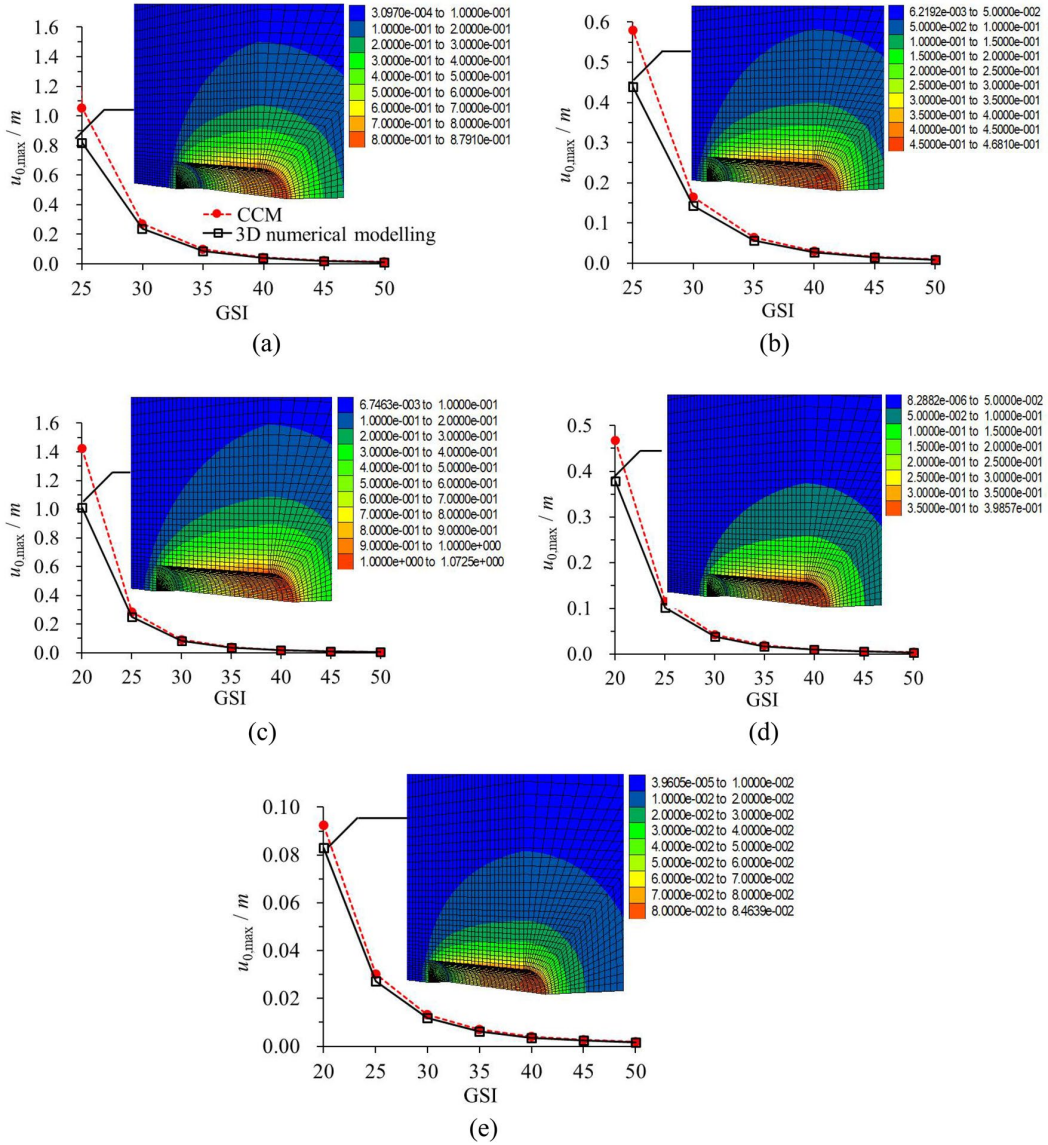
Comparison of *u*_0,max_ that solved by CCM and 3D numerical modelling^28^. (**a**) 25 MPa; (**b**) 20 MPa; (**c**) 15 MPa; (**d**) 10 MPa; (**e**) 5 MPa.

Figure 11 plots the results of *u*_0,max_ values derived from the CCM and 3D numerical modelling^29^. It is noted that in this analysis, both the 3D numerical modeling and CCM contain the process of the tunnel excavation and the liner installation. However, herein, the maximum rock deformation occurs when the liner is not installed. Essentially, *u*_0,max_ is obtained by GRC in CCM without considering the liner, and *u*_0,max_ that was solved by the 3D numerical modelling is continuously excavated without installing the liners. Therefore, in this section, the disturbance effect of the tunnel face and the group effect of the liners are not included. The difference of *u*_0,max_ is essentially influenced by the calculation discrepancy between the 2D (i.e., CCM) and 3D numerical methods (i.e., 3D numerical modelling). The aim of comparing *u*_0,max_ is to counteract the probable calculation discrepancy between the two methods.

It should be noticed that when GSI = 20, *u*_0,max_ by the CCM for *σ*_0_ = 20 MPa, 25 MPa is more than 2 m and 3 m, which would mean the total collapse of the tunnel in reality. Thus, in Fig. 11a,b, the results are not plotted for the conditions when GSI = 20. As plotted in Fig. 11, *u*_0,max_ by the CCM is higher than that by the 3D numerical modelling. The relatively smaller results in 3D numerical modelling can be attributed to the constraint of the rock zones in the longitudinal direction, or in other words, to the boundary conditions. As GSI decreases and *σ*_0_ increases, the discrepancy between the two methods grows. The maximum discrepancy occurs when GSI = 25 and *σ*_0_ = 25 MPa, for this case, *u*_0,max_ by the CCM is 28.94% higher. As GSI grows to be 30 or more, the difference becomes marginal.

When the rock deformation reaches the maximum value *u*_0,max_, the corresponding *p*_i_ is regarded as 0. While the liners are installed in the model, the liners will provide the support pressure to the rock mass, and the rock deformation should be far smaller than that when *p*_i_ = 0 with the same case. Under this condition, the discrepancy due to boundary conditions by the 2D and 3D numerical methods is negligible. If a large difference between the results obtained with CCM and 3D modelling is still observed, this should be due to other factors such as the group effect of the liners and the disturbance effect of the tunnel face.

### Comparison of initial and final rock deformations

**Figure 12:**
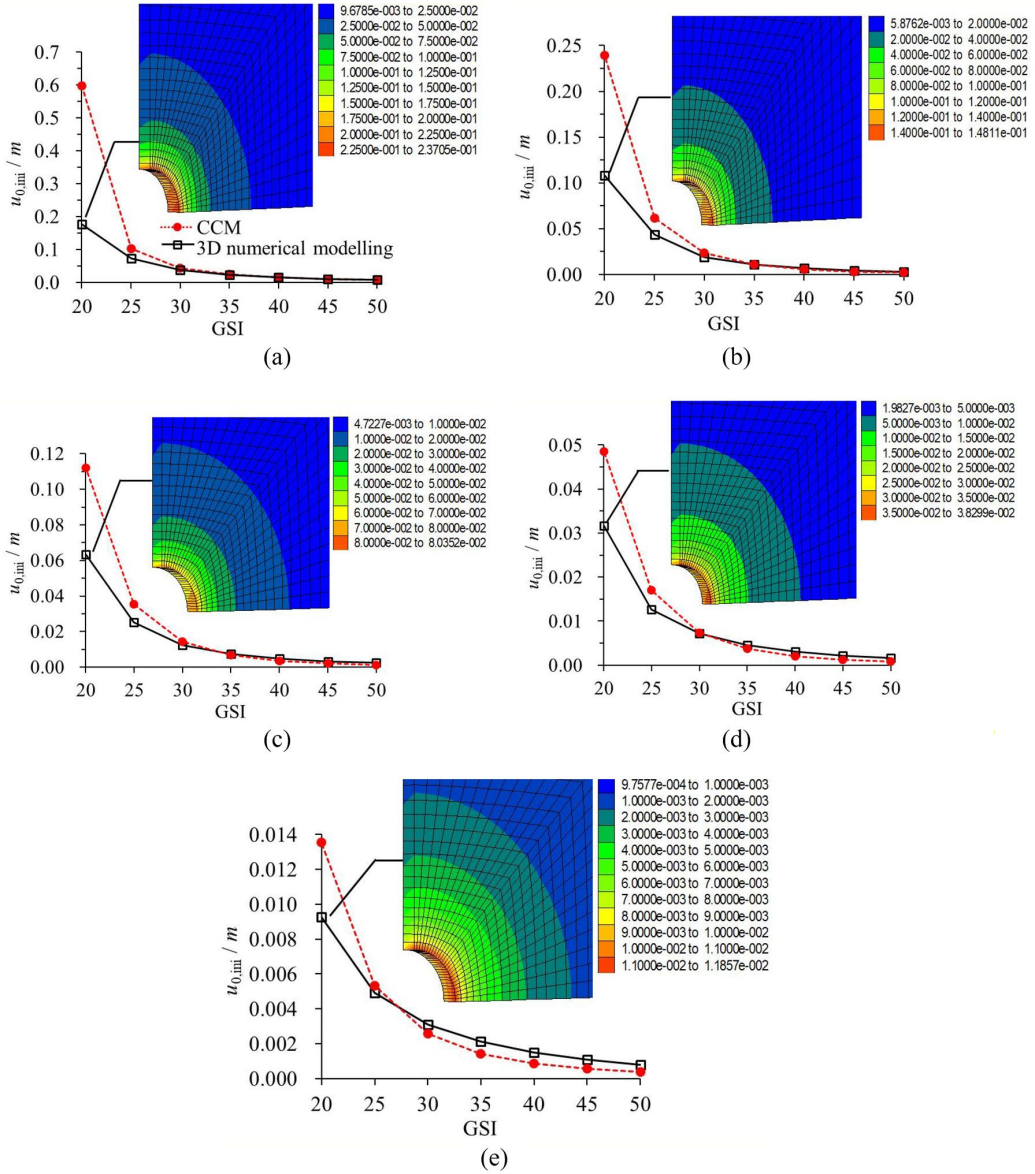
Comparison of *u*_0,ini_ that solved by CCM and 3D numerical modelling^28^. (**a**) 25 MPa; (**b**) 20 MPa; (**c**) 15 MPa; (**d**) 10 MPa; (e) 5 MPa.

**Figure 13:**
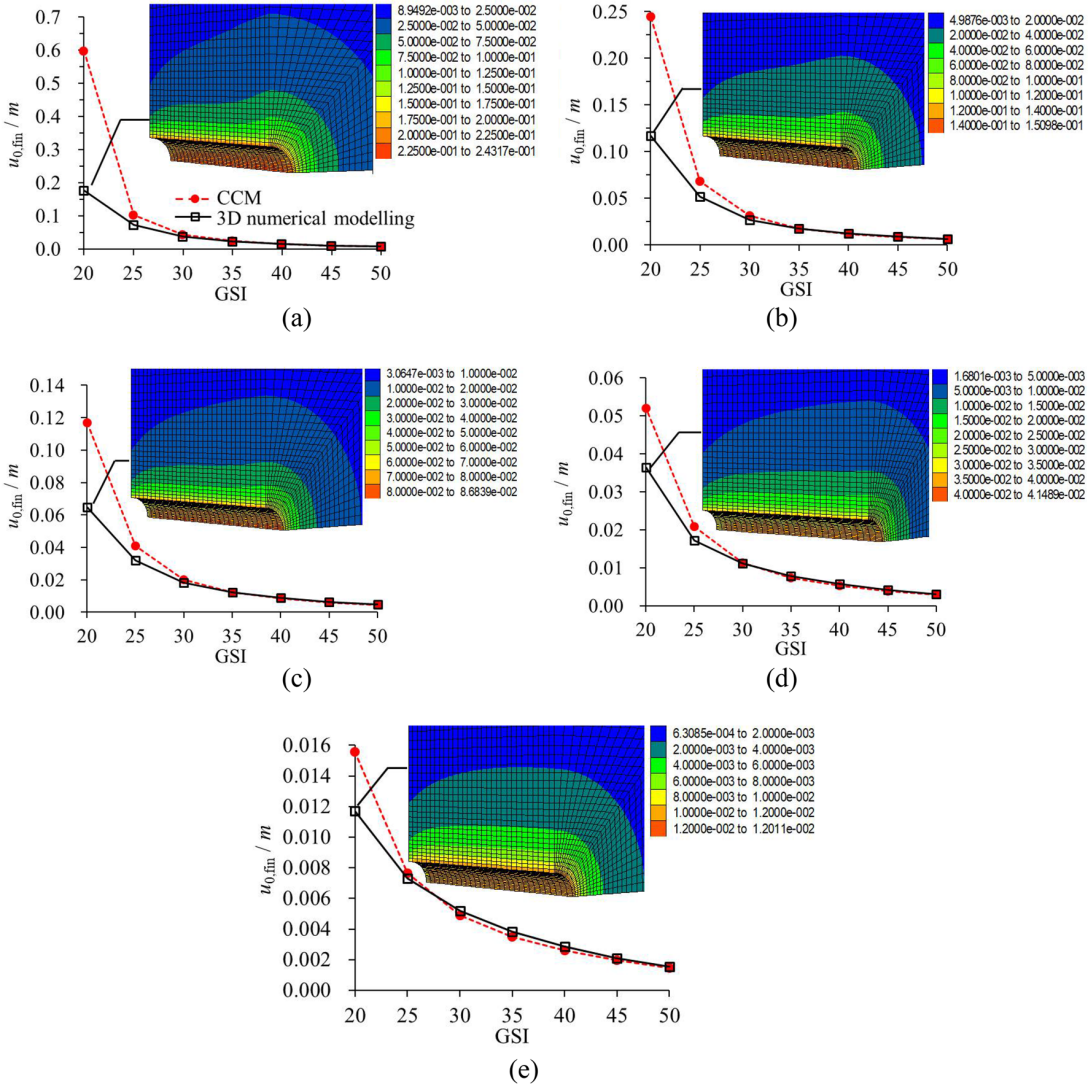
Comparison of *u*_0,fin_ that solved by CCM and 3D numerical modelling^28^. (**a**) 25 MPa; (**b**) 20 MPa; (**c**) 15 MPa; (**d**) 10 MPa; (**e**) 5 MPa.

Figures 12, 13 plot *u*_0,ini_ and *u*_0,fin_ as solved according to CCM and 3D numerical modelling^29^. Comparing Fig. 11 with Fig. 12 or Fig. 13, the difference of the rock deformation by the two methods is much higher with the liners than that without the liners. Thus, for the conditions with the liner installation, except for the numerical calculation discrepancy, the difference of the results should resort to other factors.

#### Initial rock deformation

It should be noted that, with regard to the CCM, *u*_0,ini_ is solved by the LDP, which is equal to the radial rock deformation near the tunnel face without the liner installation. In comparison, for the 3D numerical modelling, the solution of *u*_0,ini_ is based on Fig. 10b.

As shown in Fig. 12, for the poor geological condition, *u*_0,ini_ that solved by the CCM is far higher than that by the 3D numerical modelling, and a larger *σ*_0_ leads to greater discrepancy. For example, as GSI is 20, *u*_0,ini_ by CCM is 2.38 times higher than that by 3D modelling when *σ*_0_ = 25 MPa, and it is 33.09% higher than the latter when *σ*_0_ = 5 MPa. The difference of *u*_0,ini_ by the two methods declines with the increase of GSI. As GSI is 25, the difference becomes comparatively small, and as GSI grows to 30, the results by the two methods are basically equivalent. Although the deviation is minimal, it is unexpected to find that, as *σ*_0_ is under 10 MPa, and GSI surpasses 30 or more, *u*_0,ini_ that solved by CCM becomes smaller than that by the 3D numerical modelling.

To thoroughly discuss the unexpected variation of the *u*_0,ini_, Tables 3 and 4 display the initial rock deformation *u*_0,ini_ and maximum longitudinal deformation at the tunnel face *u*_l,max_ with and without the liners. It is noted that 3D numerical modelling and CCM correspond to the conditions with and without liners, respectively, and *u*_l,max_ represents the stability of the tunnel face.

**Table 3:** Initial rock deformations by 3D numerical modelling and CCM for different geological conditions.

GSI	*σ*_0_/MPa	Initial rock deformation/mm		Comparison/%
		3D numerical modelling	CCM	
50	5	0.78	0.39	100.00
50	15	2.3	1.36	69.12
50	25	4.04	2.65	52.45
25	5	4.94	5.34	− 7.49
25	25	63.77	95.51	− 33.23

**Table 4:** Maximum longitudinal deformations at the tunnel face by 3D numerical modelling and CCM for different geological conditions.

GSI	*σ*_0_/MPa	Maximum longitudinal deformation/mm		Comparison/%
		3D numerical modelling	CCM	
50	5	1.27	1.23	3.58
50	15	3.90	3.83	1.84
50	25	6.60	6.58	0.32
25	5	9.65	11.79	− 18.17
25	25	157.74	286.05	− 44.86

As displayed in Tables 3, 4, as GSI decreases and *σ*_0_ increases, the comparison of the *u*_0,max_ by the two methods is consistent with *u*_0,ini_. The consideration of the group effect of the liners and the disturbance of the tunnel face lead to the difference of deformation behaviour near the tunnel face by the two methods. With the liner, as the liner provides the support pressure to the rock mass at the tunnel periphery, on one hand, it constrains the radial rock deformation at the tunnel periphery, this relates to the group effect of the liners, but on the other hand, it prompts the longitudinal deformation to increase at the tunnel face as the forces transfers to the tunnel face, this is the disturbance of the tunnel face.

As shown in Fig. 2, for the poor geological conditions, the fictitious pressure ratio around the tunnel face is relatively small^25^, so large stress relief occurs. According to 3D numerical modelling, the liner near the tunnel face supports the rock mass at the tunnel periphery effectively. The force transferring from the liner to the tunnel face in the longitudinal direction is relatively low. Then, the group effect of liners prevails over the disturbance effect of the tunnel face. The group liners lift the rock mass near the tunnel face in comparison to the condition derived from CCM.

**Figure 14:**
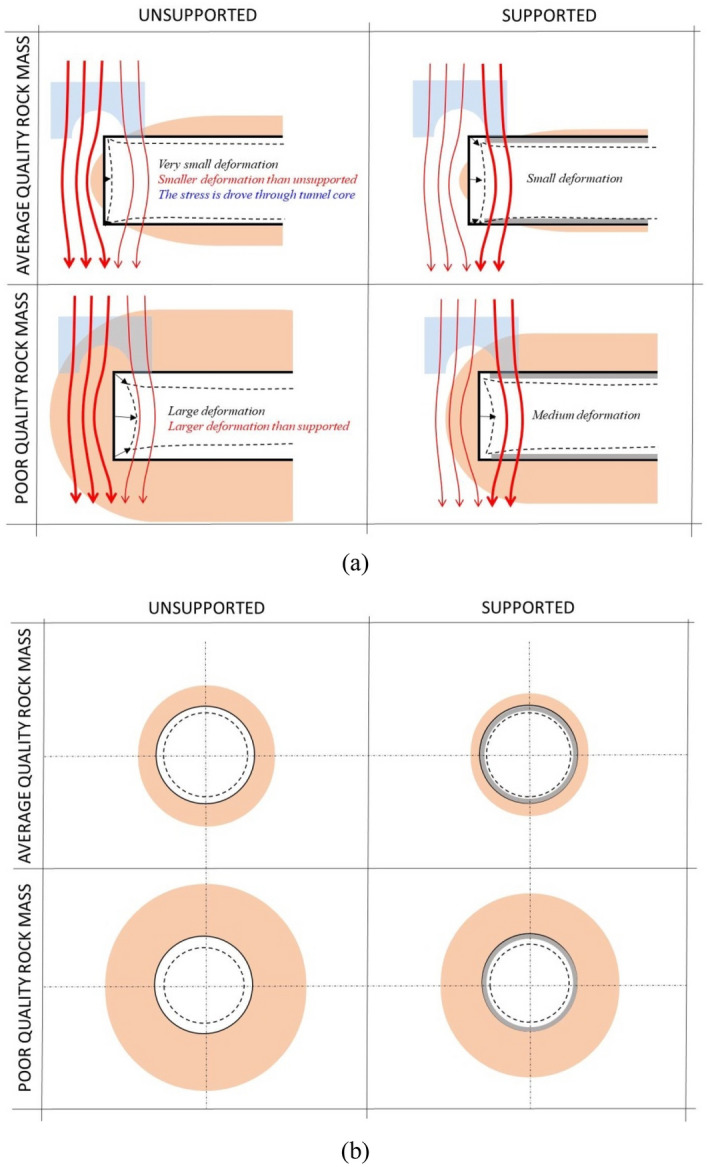
Rock deformation, load transfer and disturbance area near the tunnel face for different rock mass qualities with and without liners: (**a**) rock deformation and load transfer, (**b**) disturbance area.

Unlike for the poor rock mass quality, for better geological conditions (GSI = 50), the fictitious pressure ratio is large and the stress relief is small^25^. This implies that, around the tunnel face, even though no liner is installed, the rock mass can still be self-supporting. Hence, according to 3D numerical modelling, the performance of the liner is not as effective as for the poor rock mass with regard to decreasing the radial rock deformation. But due to the relatively large support pressure ratio, the liner near the tunnel face transfers a higher proportion of the energy to the tunnel face in the longitudinal direction. In this case, the disturbance effect of the tunnel face is greater than that of the group effect of liners. As presented in Fig. 14, because of this energy, for the 3D numerical modelling a greater deformation near the tunnel face is observed than for the CCM approach.

**Figure 15:**
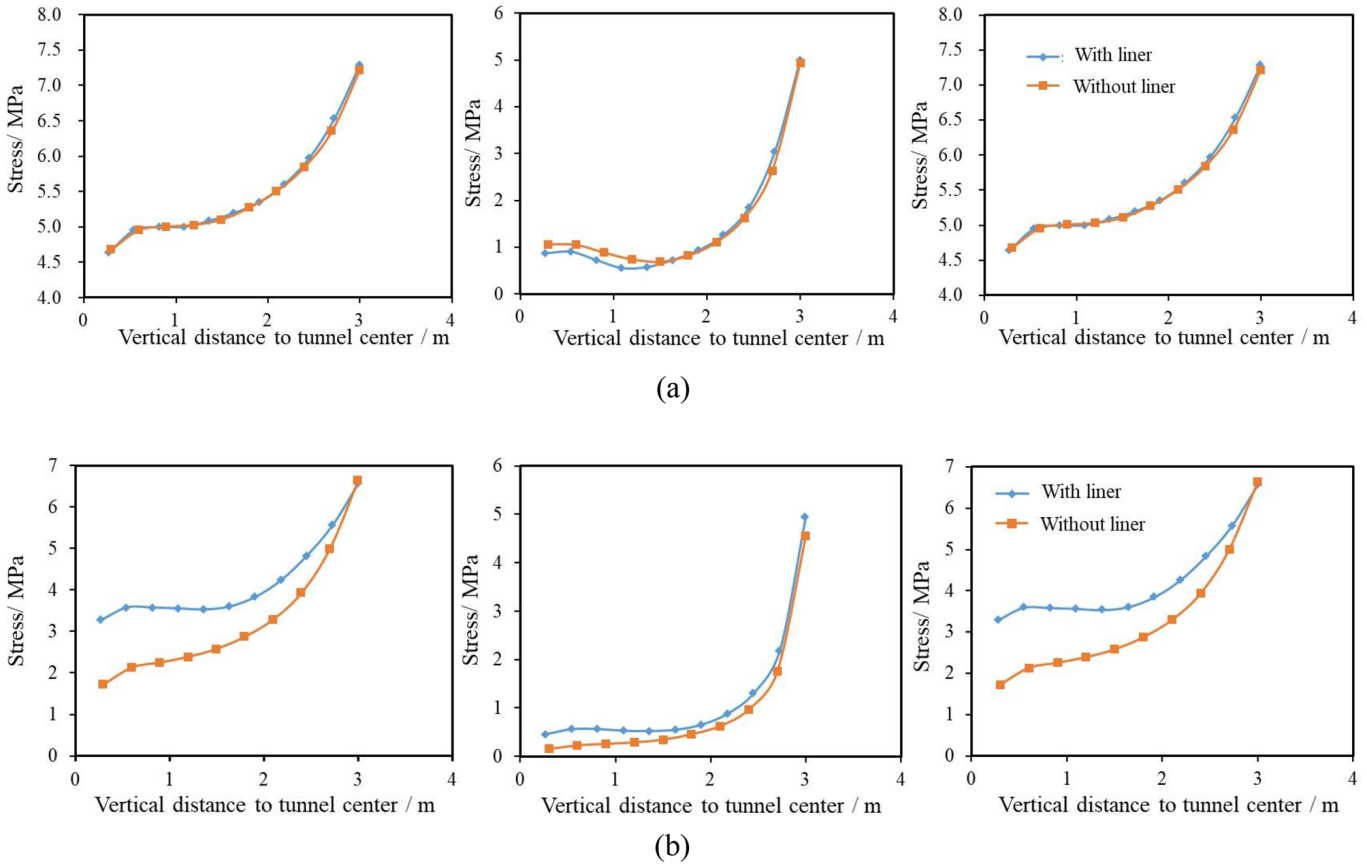
Stress distributions at the tunnel face with and without liners (**a**) GSI = 50, *σ*_0_ = 5 MPa; (**b**) GSI = 25, *σ*_0_ = 5 MPa.

As shown in Fig. 15, the above discussion can be proved by the stress distributions at the tunnel face with and without the liner. It is observed that for the poor geological condition, the stresses in the three directions with the liner are all greater than that without the liner. But the discrepancy is found to be smaller for longitudinal direction when comparing with other directions. The reason is that the liner’s effect in the radial direction is greater than in the longitudinal direction because, the large support pressure provided by the liners decreases the stress in the longitudinal direction at the tunnel face. For the good geological condition, this effect is more severe, making the stress in the longitudinal direction with the liner even slightly smaller than that without the liner.

#### Final rock deformation

As presented in Fig. 12 and Fig. 13, by comparing the *u*_0,ini_ and *u*_0,fin_, it is found that the variations of *u*_0,fin_ by the two methods are basically similar to those of *u*_0,ini_. For example, as GSI is 20, *u*_0,fin_ by CCM is 2.6 times higher than that by the 3D modelling when *σ*_0_ = 25 MPa, and it is 26.5% higher than the latter when *σ*_0_ = 5 MPa. However, for most geological conditions, the difference of *u*_0,fin_ by the two methods is not as large as that of the *u*_0,ini_. Especially for the poor geological condition, the increase from *u*_0,ini_ to *u*_0,fin_ is not obvious. For instance, when GSI is 20, the ratio of *u*_0,ini_ to *u*_0,fin_ is 79.70% and 67.44% for *σ*_0_ = 15 MPa and *σ*_0_ = 5 MPa according to the 3D numerical modelling, and it is 86.72% and 69.87% according to CCM. Therefore, for the poor geological condition, most deformation takes place near the tunnel face and before the liner installation. This is why in this squeezing conditions, it is of paramount relevance to control deformation in the tunnel face, so some tunnelling companies proposed nailing it by means of carbon fibre dowels.

### Comparison of final support pressure

#### With variable geological conditions

**Figure 16:**
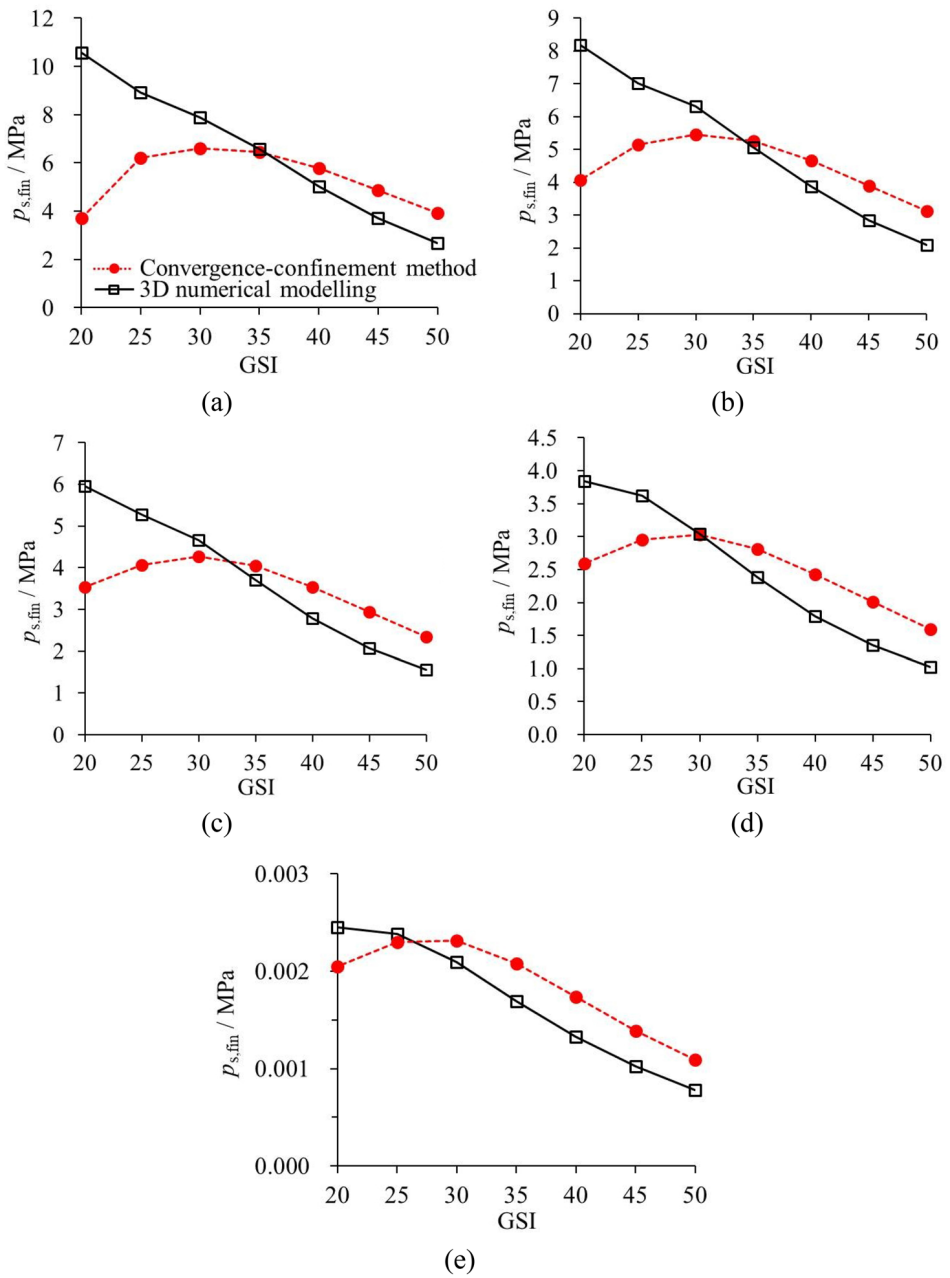
Comparison of *p*_s,fin_ that solved by CCM and 3D numerical modelling (**a**) 25 MPa; (**b**) 20 MPa; (**c**) 15 MPa; (**d**) 10 MPa; (**e**) 5 MPa.

Figure 16 plots the comparison of *p*_s,fin_ solved by means of CCM and 3D numerical modelling. A variable Δ*p*_s,fin_ is introduced to quantitatively denote the difference of CCM and 3D numerical model derived *p*_s,fin_ values,

20 $$\Delta p_{{\text{s,fin}}} \left( \% \right) = \frac{{p_{{\text{s,mol}}} - p_{{\text{s,CCM}}} }}{{p_{{\text{s,CCM}}} }}$$

where *p*_s,mol_ and *p*_s,CCM_ refers to the final support pressures derived from the 3D numerical modelling and from CCM, respectively. Table 5 displays the values of Δ*p*_s,fin_ with different analysis cases.

**Table 5:** Δ*p*_s,fin_ with different analysis cases.

Unit/%	*σ*_0_ = 25 MPa	*σ*_0_ = 20 MPa	*σ*_0_ = 15 MPa	*σ*_0_ = 10 MPa	*σ*_0_ = 5 MPa
GSI = 50	− 31.79	− 32.90	− 33.93	− 35.94	− 38.13
GSI = 45	− 23.57	− 27.08	− 29.39	− 32.31	− 35.78
GSI = 40	− 13.31	− 16.78	− 21.45	− 26.06	− 32.29
GSI = 35	1.78	− 3.72	− 8.19	− 15.19	− 26.28
GSI = 30	19.45	15.80	9.25	0.10	− 14.89
GSI = 25	43.85	35.90	29.48	22.71	5.63
GSI = 20	186.22	101.42	68.68	47.91	32.00

As observed in Table 5 and Fig. 16, for poor geological condition, when GSI is relatively small and *σ*_0_ is fairly large, CCM greatly underestimates *p*_s,fin_ in contrast to the 3D numerical modelling. For example, as GSI = 20, *p*_s,fin_ by 3D numerical modelling is 1.86 and 1.01 times larger than that by CCM for *σ*_0_ = 25 MPa and *σ*_0_ = 20 MPa, respectively. This is due to the fact that the group effect of liners is predominant. The group effect of liners represents that the liners in the 3D numerical modelling interact with other liners through load transferring. Thus, one liner at a certain section not only bears the load on it, but also the load transferred from other liners. In comparison, without consideration of the group effect of liners, one liner in CCM at certain section merely suffers from the load due to the stress relief.

For fairly good geological conditions, it is observed that *p*_s,fin_ evaluated by CCM is overestimated. As GSI = 40, 45 and 50, *p*_s,fin_ by 3D numerical modelling is 32.29%, 35.78% and 38.13% lower than that by CCM for *σ*_0_ = 5 MPa. The reason is that the disturbance effect of the tunnel face is more relevant. Because of this, the initial rock deformation is larger in 3D numerical modelling due to the greater stress relief. The remaining stress relief during the tunnel excavation becomes less relevant. This results in a smaller support pressure. For moderate geological conditions (e.g., the case when GSI = 35, *σ*_0_ = 20 MPa and when GSI = 25, *σ*_0_ = 5 MPa), the two effects are basically equivalent, which leads to similar results of *p*_s,fin_ by the CCM the 3D modelling.

#### With rigid liner and flexible liner

**Figure 17:**
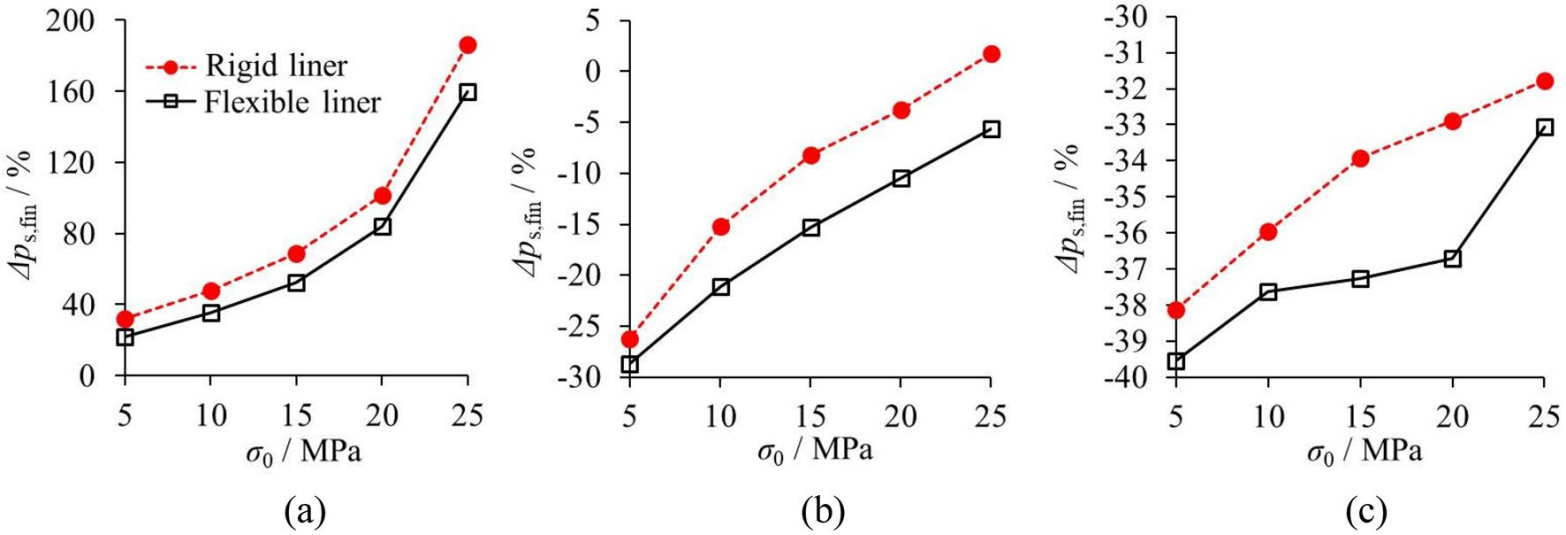
Comparison of Δ*p*_s,fin_ by CCM and 3D numerical modelling based on the rigid and flexible liners. (**a**) GSI = 20; (**b**) GSI = 35; (**c**) GSI = 50.

Figure 17 plots Δ*p*_s,fin_ by CCM and 3D numerical modelling for the rigid and flexible liners. While the rock mass is poor, the group effect of the liners is more evident than that of the disturbance effect of the tunnel face. This is the main reason why Δ*p*_s,fin_ of both the rigid and flexible liner reveals the positive value for GSI = 20. Further, since the rock deformation is significant when excavating the poor rock mass, the support performance of the rigid liner is more effective, which leads to the greater difference that solved by the CCM and 3D modelling.

In comparison, while the rock mass shows moderate or fairly good quality, the disturbance effect of the tunnel face is more evident. This leads to smaller initial rock deformation and greater liner stress for CCM than 3D numerical modelling. Hence, for GSI = 35 and GSI = 50, Δ*p*_s,fin_ tend to show the negative values. Particularly, the absolute values of the Δ*p*_s,fin_ are higher for the flexible liners. Hence, for the moderate and good geological conditions, the influence of the disturbance effect of the tunnel face on the flexible liner is greater. Furthermore, the influence of the disturbance effect of the tunnel face on the flexible liner is greater because the flexible liner is more sensitive to the change in the stress relief. Thus, the absolute values of the Δ*p*_s,fin_ are higher for the flexible liners, which implies a more significant difference between the results of both approaches.

### Comments on the support design in the application of CCM and 3D numerical modelling

The two methods, 3D numerical modelling and CCM, can be assumed as two extreme approaches regarding the impact of the 3D tunnelling effect. For the CCM, the connection of the adjacent liners is free in the longitudinal direction, and the influence of the construction sequence on the disturbance of the tunnel face is not considered. For the 3D numerical modelling herein, the adjacent liners along the longitudinal direction are rigidly connected, the effect of the construction sequence on the tunnel face is fully considered.

As the initial field stress is high and the rock quality is poor, the liner stress derived from the 3D modelling is much greater than that derived from the CCM. Then, the support design evaluated by the CCM can be unsafe. As the rock quality is moderate, the support pressures that were predicted by the two methods distinguish not that much. As the initial stress is low and the rock quality is fairly good, the support pressure that was predicted by the CCM is greater than that predicted by the 3D numerical modelling. This implies that the evaluation of support pressure by the CCM is fairly conservative.

Table 6 presents the final support pressures predicted by the two extreme cases. The support design can be predicted by combining the support pressures with the particular excavation method adopted in the practical tunnelling. It should be noticed that, the evaluation of the support pressures heavily depends on the connection rigidity among the adjacent liners and disturbance of tunnel face for the particular excavation method.

**Table 6:** *p*_s,fin_ that predicted by the CCM and 3D numerical modelling with different analysis cases.

*p*_s,fin_/MPa		Rigid liner (*t*_s_ = 0.21 m, *E*_s_ = 30 GPa)			Fexible liner (*t*_s_ = 0.18 m, *E*_s_ = 20 GPa)		
		GSI = 20	GSI = 35	GSI = 50	GSI = 20	GSI = 35	GSI = 50
*σ*_0_ = 25 MPa	CCM	3.70	6.45	3.92	3.68	5.31	2.63
	3D modelling	10.58	6.56	2.67	9.57	5.01	1.76
*σ*_0_ = 20 MPa	CCM	4.07	5.26	3.13	4.02	4.23	2.07
	3D modelling	8.19	5.06	2.10	7.38	3.78	1.31
*σ*_0_ = 15 MPa	CCM	3.53	4.05	2.35	3.45	3.16	1.52
	3D modelling	5.96	3.72	1.55	5.27	2.68	0.96
*σ*_0_ = 10 MPa	CCM	2.60	2.82	1.60	2.50	2.11	1.01
	3D modelling	3.84	2.39	1.03	3.38	1.67	0.63
*σ*_0_ = 5 MPa	CCM	1.54	1.56	0.82	1.42	1.09	0.51
	3D modelling	2.03	1.15	0.51	1.73	0.78	0.31

## Conclusions

This paper mainly discusses the limitation of CCM by comparing the results obtained by the CCM and 3D numerical modelling. A procedure for obtaining the initial rock deformation, final rock deformation, support pressure and liner stress by CCM is proposed. These variables are also obtained by the 3D numerical modelling. The results of the two methods are compared.

By comparing these results, the limitation of CCM can be attributed to the group effect of liners and the disturbance effect of the tunnel face. The group effect of liners, which is ignored by the CCM, denotes the interactions of the adjacent liners along the longitudinal direction in the 3D numerical modelling or in practical engineering. Due to the group effect, the load from the tunnel advance is shared with by the adjacent liners, which lifts the rock mass; and simultaneously, one liner at certain section not only bears the load on it, but also the load transferred from other liners. Therefore, according to CCM, the group effect of liners induces relatively small rock deformation and large support pressure in comparison to the 3D numerical modelling.

The disturbance effect of the tunnel face is a reflection of the stress relief due to the tunnelling excavation. Because of the more complex excavation sequence, the disturbance effect in the 3D numerical modelling is more severe than that in the CCM. This effect makes the rock deformation around the tunnel face by the 3D numerical modelling higher than that derived from CCM. Due to the larger initial rock deformation, the remaining stress relief on the liners during the tunnel excavation becomes smaller, which results in smaller support pressure in the 3D numerical modelling.

Both effects (i.e., the group effect of liners and the disturbance effect of the tunnel face) exist among all the analysis cases, whereas their significance is variable. For the poor geological condition, the group effect of liners is more remarkable; and on the contrary, for good quality condition, the group effect of liners is not as obvious as the disturbance effect of the tunnel face. Although the disturbance effect of the tunnel face prevails for certain conditions, the group effect of liners is significant because it leads to relevant differences in the results of the two methods for the poor rock mass conditions, which should be highlighted.

The comparison of the support design by the CCM and 3D numerical modelling under different geological conditions is presented. As the initial field stress is significant and the rock quality is poor, the liner stress solved by the 3D modelling is much greater than that by the CCM. Then, the support design evaluated by the CCM is significantly unsafe. As the rock quality is moderate, the support pressures predicted by both methods are similar. As the initial stress is low and the rock quality is fairly good, the support pressure predicted by the CCM is greater than that predicted by the 3D numerical modelling. This implies the evaluation of support pressure by the CCM is fairly conservative in this case.

## Supplementary Information

Supplementary Information 1.

Supplementary Information 2.

## Data Availability

The datasets used and/or analysed during the current study available from the corresponding author on reasonable request.

## Abbreviations

CCM: Convergence-confinement method
2D: Two-dimensional
LDP: Longitudinal deformation profile
*x*: Distance to the tunnel face
*u*_0,fin_: The equilibrium of deformation for a final value corresponding to the intersection point between SCC and GRC.
*p*_fic_: Fictitious pressure
*σ*_r_: Radial stress
*ε*_r_: Radial strain
*σ*_1_,: Major principal stress
*u*: Rock deformation in the radial direction
*m*_b_,* s*, *a*: Strength parameters of the Hoek–Brown rock mass
$\sigma_{{{\text{r}}2}}$: Radial stress at the elastic–plastic boundary
*u*_0_: Rock deformation at the tunnel periphery
$\varepsilon_{{{\text{r(}}i)}}$,$\varepsilon_{{{\uptheta }\left( i \right)}}$: Radial and tangential strains at *r* = *r*_(__*i*__)_
$\varepsilon_{{{\text{r}}(i - 1)}}$,$\varepsilon_{{{\uptheta }(i - 1)}}$: Radial and tangential strains at *r* = *r*_(__*i*__−1)_
*σ*_r__(__*i*__)_,*σ*_r__(__*i*__−1)_: Radial stresses at the inner and outer boundaries of each annulus
Δ*σ*_r_: Constant radial stress increment
*p*_s__,fin_: Final support pressure
*u*^*^,* X*^*^ and *R*^*^: Initial rock deformations at the tunnel periphery, the distance to the tunnel face and the radius of the plastic zone.
*K*_s_: Stiffness of the liner
*μ*_s_: Poisson’s ratio of the liner
*ψ*: Dilatancy angle
*p*_s,CCM_: Final support pressure derived from from CCM
3D: Three-dimensional
GRC: Ground reaction curve
SCC: Support characteristic curve
*u*_0,ini_: Initial rock deformation, i.e., certain amount of deformation that the rock mass has already experienced. In this analysis, the initial rock deformation is assumed as the rock deformation of the tunnel periphery at the tunnel face
*f*_0_: Stress relief during tunnelling
*σ*_0_: Initial stress
*σ*_θ_: Tangential stress
ε_θ_: Tangential strain
*σ*_3_: Minor principal stress
*r*: Distance within the rock mass to the center of the circular opening
*σ*_ci_: Uniaxial compression strength of intact rock
*R*_p_: Radius of plastic zone
*p*_i_: Internal pressure
*φ*: Friction angle
*r*(*i*), *r*(*i*−1): Radii of the inner and outer boundaries of the ith annulus
*n*: Total number of the concentric annuli
*u*_r(__*i*__)_: Radial displacement at *r* = *r*(*i*)
*μ*: Poisson’s ratio
*u*_0,max_: Maximum rock deformation at the tunnel periphery when the internal pressure is 0
*E*_s_: Elastic modulus of the liner
*t*_s_: Thickness of the liner
*p*_s,mol_: Final support pressure derived from the 3D numerical modelling
Δ*p*_s,fin_: Difference of CCM and 3D numerical model derived* p*_s,fin_ values
