# Supplementary material for: Limitation of convergence-confinement method on three-dimensional tunnelling effect
Source: Sci Rep. 2023 Feb 3;13:1988. doi: 10.1038/s41598-023-29062-5 (PMC9898246; doi:10.1038/s41598-023-29062-5)
Supplement: Supplementary file 1 — Supplementary Information 1. [file 41598_2023_29062_MOESM1_ESM.docx]

**Appendix A**

The variables in Eqs. (7) and (8) are:

$A_{(i-1)}\text{=}\varepsilon_{\text{r(}i-1\text{)}}\text{+}K_{\text{ψ}}\varepsilon_{\text{θ(}i-1\text{)}}+\left(1+\mu\right)\left[\Delta\sigma_{\text{r}}\left(1-\mu-K_{\text{ψ}}\mu\right)+B_{(i-1)}\left(K_{\text{ψ}}-K_{\text{ψ}}\mu-\mu\right)\right]/E_{r}$ (A1)

$B_{(i-1)}\text{=}\Delta\sigma_{\text{r}}\text{+}H\left(\sigma_{\text{r}\left(i\right)}\right)-H\left(\sigma_{\text{r}\left(i-1\right)}\right)$ (A2)

 (A3)

 (A4)

 (A5)

The initial values at *r* = *r*_(0)_ are:

 (A6)

 (A7)

 (A8)

Eqs. (7) and (8) are obtained through the Eqs. (A1) to (A8) step by step.
