# Supplementary material for: Limitation of convergence-confinement method on three-dimensional tunnelling effect
Source: Sci Rep. 2023 Feb 3;13:1988. doi: 10.1038/s41598-023-29062-5 (PMC9898246; doi:10.1038/s41598-023-29062-5)
Supplement: Supplementary file 2 — Supplementary Information 2. [file 41598_2023_29062_MOESM2_ESM.docx]

**Appendix B**

The coefficients in the equation include *a*_1_*、b*_1_*、b*_2_*、c*_1_*、c*_2_*、c*_3_*、d*_1_*、d*_2_*、d*_3_*、d*_4_*、e*_1_*、e*_2_*、e*_3_*、e*_4_*、e*_5_*.* The coefficients are presented in Table B1.

Table B1 Coefficients in fitting equation

| *X*^*^＞0 | | | | | | | |
| --- | --- | --- | --- | --- | --- | --- | --- |
| *a*_1_ | -0.4559 | *c*_1_ | -0.2961 | *d*_1_ | 0.0496 | *e*_1_ | -0.0029 |
| *b*_1_ | 0.6861 | *c*_2_ | 0.1099 | *d*_2_ | -0.0091 | *e*_2_ | 2.0167×10^-4^ |
| *b*_2_ | -0.4168 | *c*_3_ | 0.0375 | *d*_3_ | -0.0082 | *e*_3_ | 3.763×10^-4^ |
|  |  |  |  | *d*_4_ | -0.0021 | *e*_4_ | 1.909×10^-4^ |
|  |  |  |  |  |  | *e*_5_ | 5.032×10^-5^ |
| *R*^2^ = 99.05 % | | | | | | | |
